# Methionine oxidation of CLK4 promotes the metabolic switch and redox homeostasis in esophageal carcinoma via inhibiting MITF selective autophagy

**DOI:** 10.1002/ctm2.719

**Published:** 2022-01-29

**Authors:** Yaxing Shen, Heng Zhang, Shihua Yao, Feng Su, Hao Wang, Jun Yin, Yong Fang, Lijie Tan, Kaiguang Zhang, Xiangshan Fan, Ming Zhong, Qingxin Zhou, Jie He, Zhiyong Zhang

**Affiliations:** ^1^ Department of Thoracic Surgery Zhongshan Hospital, Fudan University Shanghai China; ^2^ Department of Thoracic Surgery, National Cancer Center/National Clinical Research Center for Cancer/Cancer Hospital Chinese Academy of Medical Sciences and Peking Union Medical College Beijing China; ^3^ Department of Histology and Embryology Xiang Ya School of Medicine, Central South University Changsha China; ^4^ Department of Thoracic Surgery Navy Military Medical University Affiliated Changhai Hospital Shanghai China; ^5^ Department of Digestive Disease The First Affiliated Hospital of USTC (Anhui Provincial Hospital) Hefei China; ^6^ Department of Pathology The affiliated Drum Tower Hospital, Nanjing University Medical School Nanjing China; ^7^ Department of Critical Care Medicine Zhongshan Hospital, Fudan University Shanghai China; ^8^ Department of Gastrointestinal Oncology Harbin Medical University Cancer Hospital Harbin China; ^9^ National Center for International Research of Biological Targeting Diagnosis and Therapy (Guangxi Key Laboratory of Biological Targeting Diagnosis and Therapy Research) Guangxi Medical University Nanning China; ^10^ Department of Surgery, Robert‐Wood‐Johnson Medical School University Hospital Rutgers University, State University of New Jersey New Brunswick New Jersey USA

**Keywords:** CDC‐like kinase 4, esophageal squamous cell carcinoma, methionine oxidation, microphthalmia‐associated transcription factor, purine synthesis

## Abstract

**Background:**

Metabolic reprogramming and redox homeostasis contribute to esophageal squamous cell carcinoma (ESCC). CDC‐like kinase 4 (CLK4) is a dual‐specificity kinase that can phosphorylate substrates’ tyrosine or serine/threonine residue. However, the role and mechanism of CLK4 in ESCC remain unknown.

**Methods:**

CLK4 expression was analysed using publicly available datasets and confirmed in ESCC tissues and cell lines. The biological roles of CLK4 were studied with gain and loss‐of‐function experiments. Mass spectrometry was employed to examine the effects of CLK4 on metabolic profiling. In vitro kinase assay, co‐immunoprecipitation, glutathione S‐transferase pulldown, chromatin immunoprecipitation and luciferase reporter were used to elucidate the relationship among CLK4, microphthalmia‐associated transcription factor (MITF), COP1 and ZRANB1.

**Results:**

CLK4 down‐regulation was observed in ESCC cell lines and clinical samples and associated with the methylation of its promoter. Low levels of CLK4 promoted ESCC development by affecting the purine synthesis pathway and nicotinamide adenine dinucleotide phosphate (NADPH)/nicotinamide adenine dinucleotide phosphate (NADP^+^) ratio. Interestingly, CLK4 inhibited ESCC development by blocking MITF‐enhanced de novo purine synthesis and redox balance. Mechanistically, wild type CLK4 (WT‐CLK4) but not kinase‐dead CLK4‐K189R mutant phosphorylated MITF at Y360. This modification promoted its interaction with E3 ligase COP1 and its K63‐linked ubiquitination at K308/K372, leading to sequestosome 1 recognition and autophagic degradation. However, the deubiquitinase ZRANB1 rescued MITF ubiquitination and degradation. In turn, MITF bound to E‐ rather than M‐boxes in *CLK4* promoter and transcriptionally down‐regulated its expression in ESCC. Clinically, the negative correlations were observed between CLK4, MITF, and purine metabolic markers, which predicts a poor clinical outcome of ESCC patients. Notably, CLK4 itself was a redox‐sensitive kinase, and its methionine oxidation at M307 impaired kinase activity, enhanced mitochondria length and inhibited lipid peroxidation, contributing to ESCC.

**Conclusions:**

Our data highlight the potential role of CLK4 in modulating redox status and nucleotide metabolism, suggesting potential therapeutic targets in ESCC treatment.

## Graphical abstract

1. CLK4 is markedly down‐regulated in ESCC due to the methylation of its promoter.

2. CLK4 inhibits ESCC development by blocking MITF‐enhanced nucleotide metabolism and redox homeostasis.

3. CLK4‐mediated MITF Y360 phosphorylation dictates MITF selective autophagic degradation.

4. CLK4 itself is a redox‐sensitive kinase, and its methionine oxidation at M307 impairs kinase activity and contributes to ESCC.

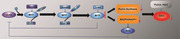

## 1 BACKGROUND

Esophageal cancer (EC) is the sixth lethal neoplasm worldwide.^1^ Although recent advancements in surgical techniques and adjuvant therapies have significantly improved overall survival, the prognosis of patients with advanced EC remains poor. EC exhibits two main histologic subtypes, esophageal adenocarcinoma and esophageal squamous cell carcinoma (ESCC), which differ significantly in molecular alterations, affected populations, associated risk factors, and response to treatment.^2^ ESCC also exhibits high intra‐tumoral molecular and mutational heterogeneity rates, thereby limiting therapy efficacy and challenging to ESCC therapy.^3^ So far, no molecular targeting agent has shown apparent effectiveness in ESCC. Thus, a better understanding of the mechanisms underlying ESCC development may provide critical information for predicting the prognosis and improving therapy for patients.

Cdc2‐like kinase 4 (CLK4) belongs to the LAMMER kinase family, including three other characterized isoforms CLK1‐3^4^. Because a well‐known target of CLK family kinases (CLKs) is a serine‐ and arginine‐rich splicing protein of the spliceosome,^4^ this family can regulate the alternative splicing of β‐globin, Tau, and PKCβII pre‐mRNA.^5^ In addition, the CLKs can phosphorylate exogenous proteins or lead to auto‐phosphorylation itself at threonine, serine, or tyrosine residue.^6^ Recent studies reported that CLK1‐3 contributed to carcinogensis.^7^ However, very little was known about the role of CLK4 in tumors. So far, CLK4 is the most poorly characterized member of the CLK family. Although the CLKs share sequence homology, CLK4 appears to be the only family member interacting with UBL5, suggesting that CLK4 may have a role distinct from the other CLKs.^8^

In this study, we found that the methylation of CLK4 promoter significantly down‐regulated the levels of CLK4, which is correlated with the progression of ESCC and poor prognosis. We further demonstrated that microphthalmia‐associated transcription factor (MITF)‐induced purine synthesis and redox status were the significant phenotypes of CLK4 knockdown in ESCC cells. In turn, redox signaling regulated CLK4 activity. Together, these data may suggest new therapeutic strategies for ESCC patients with dysregulated CLK4 expression.

## 2 METHODS

### 2.1 Cell lines, antibodies and reagents

HEK293T cells (from ATCC CRL‐3216), a human papillomavirus E6/E7/telomeraseimmortalized normal esophageal epithelial cell line (NE1) (as normal esophageal epithelial control) and several ESCC cell lines derived from humans (KYSE30, Eca109, KYSE510, Ec9706, KYSE520, KYSE140 and KYSE150) were obtained from the Cell Culture Center of Peking Union Medical College. These cell lines were kept using DMEM or RPMI1640 bought from Gibco, in which fetal bovine serum (10%) from Sigma was added. But a defined keratinocyte serum‐free medium was used to culture NE1 cells along with EpiLife medium with 60‐μM calcium (Invitrogen) and growth supplements at a 1:1 mixture. Protease inhibitor cocktail (Cat#: sc‐45045) and MG‐132 (Cat#:1211877‐36‐9) were purchased from Santa Cruz. ^14^C‐Glycine, ^13^C‐glycine, Tween 20, cycloheximide (CHX), Sodium vanadate and X‐film were from Millipore and Sigma‐Aldrich, respectively. Lipofectamine 2000, HEPES, phosphate buffered saline (PBS), glutamine and antibiotics were purchased from Invitrogen. Site‐Directed Mutagenesis Kit (Catalog # 210513 and #210515) was obtained from Agilent. The antibodies were used in this project as follows: anti‐glutathione S‐transferase (GST) antibody (Sigma, #G7781‐100UL and Abcam #ab19256), anti‐His (Sigma, #SAB4301134‐100UL and Abcam, #ab18184), CLK4 (Abcam, #ab67936), anti‐Flag M2 (Sigma, F3165‐2MG), CLK3 (Santa Cruz Biotechnology, #sc‐365225), glyceraldehyde phosphate dehydrogenase (GAPDH) (Sigma, G8709 and Abcam, #ab9485), PPAT (MyBioSource, #MBS2539826 and Abcam, #ab204366), Ki‐67 (Sigma, #Cat: 100130‐MM22 and Cell Signaling, #9129), USP13 (Santa Cruz, sc‐390316), optineurin (OPTN) (Cell Signaling, #70928), sequestosome 1 (SQSTM1) (Cell Signaling, #23214), NBR1 (Abcam, #ab55474), NDP52 (Abcam, #ab68588), BCL2/adenovirus E1B 19 kDa interacting protein 3‐like (NIX)) (Abcam, #ab8399), toll interacting protein (Tollip) (Sigma, #HPA038261), anti‐hemagglutinin (HA) (Sigma, #H6908‐100UL), MITF (Abcam, #ab20663), anti‐green fluorescent protein (GFP) (Abcam, #ab290), anti‐V5 (Sigma, #V8137‐.2MG). Anti‐MYC protooncogene (A190‐205A) and anti‐tubulin (A302‐630A) were purchased from Bethyl Laboratories. Anti‐ubiquitin Lys63‐specific antibody (no. 2210353) was from Millipore. Other antibodies were as follows: anti‐ZRANB1 antibody (Abcam, #ab103417), phosphoserine/threonine antibody (Abcam, #ab117253), tubulin (Sigma, #T0926), 4G10 phosphotyrosine antibody (Sigma, #05‐321), β‐actin (Sigma, #A5441). Secondary antibodies were obtained from Bio‐Rad (#1706516 and #1706515) and ThermoFisher (Cat # G‐21234). And this group made a specific p‐MITF‐Y360 antibody through one similar method described previously.^7^

### 2.2 Human ESCC samples

Human ESCC Clinical sample cohorts were from the affiliated hospitals, Fudan University and Harbin Medical University, all of which were enrolled. And all participants involved in this project received one written informed consent. The institutional ethical review board from the University‐affiliated Hospital approved this project.

### 2.3 Tissue array and immunohistochemistry

The formalin‐fixed samples, including non‐tumor and ESCC tumor or tissue array purchased from ALPHELYS, were used. Every sample's section (4 μm) was deparaffinized, autoclaved in ethylenediaminetetraacetic acid buffer. Then at room temperature, fresh 3% H_2_O_2_ was used to treat these sections in methanol for about 10 min. Next, indicated primary antibodies or PBS as negative control were added, and overnight incubation was carried out at 4 °C. Then rinsed the tissue section 5 min three times with PBS and incubated secondary antibodies for 30 min at room temperature. Finally, 3,3‐diaminobenzidine was used, and counterstaining was performed using hematoxylin for visualization.

### 2.4 Deoxyribonucleic acid (DNA) methylation analysis of CLK4 promoter

CpG islands were detected in the promoter of CLK4 by utilizing the openly accessible MethPrimer. With the help of the SMART, a web tool, we investigated DNA methylation profile, survival curve and correlation analysis of the Cancer Genome Atlas (TCGA) Esophageal carcinoma (ESCA) project, which are related to the *CLK4* gene. The website (http://www.bioinfo‐zs.com/smartapp/) is also openly accessible.

### 2.5 Metabolomics analysis

The indicated samples of ESCC were clarified by spinning. As performed previously,^7^ the supernatant was analysed to elucidate the metabolomics through liquid chromatography with tandem mass spectrometry (LC‐MS/MS). PeakView^TM^ software with XIC Manager 1.2.0 was from ABSciex, which was used to process the raw data for peak picking. According to IROA standards (IROA Technology), the metabolites were identified. Finally, MultiQuant 3.0.2 software was used to get the relative values of metabolites, which are evaluated with Holm false discovery rate (FDR)‐correction and Fisher's post hoc analysis following a one‐way ANOVA. ^14^CO_2_ release of ESCC cells was monitored to analyse oxidative pentose phosphate pathway (PPP) flux. And the ratios of NADPH/NADP^+^ were determined by measuring ESCC cells at 565 nm on the basis of the protocol (BioAssay Systems). Glutathione (GSH) measure and lipid peroxidation were performed as described elsewhere.^9^

### 2.6 Ubiquitination and deubiquitination assays

As described before,^10^ indicated constructs were co‐transfected into HEK293 cells HA‐Ub or HA‐K63Ub with or without ZRANB1 or USP13, respectively. Forty‐eight hours later, co‐immunoprecipitation (co‐IP) and western blots were performed with indicated antibodies.

### 2.7 In vitro kinase assay

As described previously,^11^ bacterially expressed and purified recombinant CLK4 (.5 μg) was incubated with recombinant MITF (.5 μg) in 40 ml of kinase buffer, which contains 2 mM dithiothreitol (DTT), 1 mM NaVO4, 40 mM HEPES‐KOH, pH 7.8, 12 mMβ‐glycerophosphate, 10 mM MgCl_2_, and 1 mM EGTA. The reaction was carried out along with (γ‐^32^P) ATP (10 μCi) at 30°C. Thirty minutes later, added an equal volume (40 ml) of 2 × sodium dodecyl sulfate (SDS) sample buffer to stop the reaction. Then the samples were subjected to autography for detecting phosphorylation signals.

### 2.8 Western blot and co‐IP

Briefly, at 4°C, whole proteins from cells or homogenized tumor or non‐tumor tissues were lysed with radioimmunoprecipitation assay buffer, in which protease and phosphatase inhibitors or vanadate were added.^11^ After applied to SDS–polyacrylamide gel electrophoresis (PAGE) gels, the samples were transferred to polyvinylidene difluoride membranes (ThermoFisher Scientific). Tris‐buffered saline, 0.1% Tween 20 (TBST) buffer containing 5% milk was used to block. One hour later, incubated the membrane with the indicated primary antibody at 4°C for 24 h. Then membranes were washed using TBST buffer, peroxidase‐conjugated secondary antibody (Bio‐Rad) was used and incubated at room temperature for about 1–2 h. Finally, Pierce ECL (ThermoFisher Scientific) was used to detect bands.

For co‐IP analysis, as described previously,^12^ .8 mg of cell extracts lysed with buffer containing 1% NP‐40 were pre‐cleared. Then the samples were incubated with IgG as control or indicated primary antibodies overnight with constant rotation at 4°C. Protein G magnetic beads (Santa Cruz) were added and incubated for 2 h. The proteins were eluted from beads and boiled in the loading buffer of SDS‐PAGE (4x) for 5 min. Finally, immunoblots were performed using the antibodies as described.

### 2.9 LC‐MS/MS

HEK293T or indicated ESCC cells were stably expressed with indicated constructs. On the basis of the manufacturer's protocols (ThermoFisher Scientific), the immune‐precipitated complex or specifically treated samples were assayed at Shanghai Mass Spectrometry and Proteomics Facility.

### 2.10 cDNA cloning and reverse transcriptase PCR analysis

cDNA sequences encoding HA‐ and Myc‐tagged human COP1 were synthesized by GenScript and cloned into the retroviral vector, pMIG‐w, by PCR using the high‐fidelity PrimeSTAR Max DNA Polymerase (Takara). The ligase‐dead COP1 mutant (C156/C163A) was made through the QuikChange Lightning Multi Site‐Directed Mutagenesis Kit (Agilent) and then cloned into the retroviral pMIG‐w vector. ZRANB1 and its inactive point mutants were generated as previously described.^13^ Dr. Wang (The State University of New Jersey) nicely provided HA‐WT‐ub, K63R‐ub and K48R‐ub as gifts. K48‐ub, USP13, MITF and K63‐ub constructs were purchased from Addgene. CLK3 and CLK4 cDNAs were obtained by revers trasncriptase PCR (RT‐PCR) as described previously.^7^, ^14^ The constructs were transduced into HEK293T cells or ESCC cells through Lipofectamine 2000 (Invitrogen). FuGENE 6 kit (Promega) was also used to establish overexpression cell lines based on the protocol. *CLK3* primers were as follows: *CLK3* (5′‐ATGCATCACTGTAAGCGATACCG‐3′ and 5′‐AAGCACTCCACCACCTTGCCAAA‐3′). And 94°C (30 sec), 55°C (1 min), 72°C (1 min), then repeating at least 30 cycles, which was for *CLK3* PCR conditions. *CLK4* primers are: (5′‐CGGAATTCATGCGGCATTCCAAACGAACTC‐3′ and 5′‐GCA CTCTACAACTTTGCCAAAGGC‐3′). 94°C (30 sec), 65°C (1 min), 72°C (1 min), then repeating at least 32 cycles, which was for *CLK4* PCR conditions. The mutagenesis of CLK4‐K189R and MITF‐Y360 F was generated as in Zhu et al.^13^ All wild type (WT)‐constructs and mutants were confirmed using sequencing assays. For real‐time (RT)‐PCR assays, ribonucleic acid (RNA) was extracted using TRIzol (Invitrogen) and phenol/chloroform, and the process of reverse transcription was carried out with Super Script III One Step reverse transcriptase PCR System with Platinum *Taq* Kit (Invitrogen). The products of RT‐PCR were examined through 1% agarose gels. GAPDH functioned as control, and its primers were: 5′‐GCCCAATACGACCAAATCC‐3′ and 5′‐CACCACATCGCTCAGACAC‐3′. The data analysis was performed using the comparative 2^−ΔCT^ method from three independent experiments. All of these primers for quantitative reverse transcriptase PCR (qRT‐PCR) were purchased from Life Technology.

### 2.11 siRNA or shRNA or construct transfection

Knockdown of target genes such as *MITF, CLK4* and *COP1* by siRNA was executed via Lipofectamine 2000 based on the protocol, and scramble transfection acted as a negative control. The knockdown of *CLK4*, *ZRANB1, ATG5*, *SQSTM1*, *BECN1* and *OPTN* using shRNA was carried out based on the protocol from Santa Cruz Biotechnologies, Invitrogen and Sigma, respectively. Briefly, ESCC cells were transduced with shRNA against *CLK4* (#1 and #2), or shcontrol with lentivirus particles at multiplicity of infection about 1 in the presence of polybrene (5 mg/ml). After two weeks of puromycin (2 mg/ml) selection, stable polyclonal ESCC cell lines with *shcontrol* or *shCLK4* were established. The efficiency of knockdown was confirmed using qRT‐PCR and Western blots. Their sequences were as follows: *shCLK4*#1:5′‐GCAAACCGUUGAAGGAAUU‐3′; *shCLK4*#2:5′‐ AAAGCGGGTATCGAATCCU‐3′; or *shcontrol*: 5′‐AATGCTCGCACAGCACAAG‐3′; *siMITF*: 5′‐UAGAGGUCGAUCAAGUUUCCA‐3′ and 5′‐GAAACUUGAUCGACCUCUACA‐3′;*scramble*: ′, 5′‐UGGUUUACAUGUCGACUAA‐3′ and 5′‐UAAGGCUAUGAAGAGAUAC‐3′. *siCOP1*: 5′‐GGCUUAUACUCUCCUGUCA‐3′ and 5′‐UGACAGGAGAGUAUAAGCC‐3′; *siCLK4*: 5′ ‐GCAAACCGUUGAAGGAAUU‐3′. Its scramble was also purchased from Invitrogen. *shATG5*‐#1: 5′‐GCCATCAATCGGAAACTCATGGAATATCC‐3′; *shATG5*‐#2: 5′‐GCCTGTCAAATCATAGTAT‐3′; *shSQSTM1*: 5′‐TAGTACAACTGCTAGTTATTT‐3′; targeting sequence for *BECN1* is 5′‐CCGACTTGTTCCTTACGGAAA‐3′; for *OPTN*: 5′‐GCACGGCATCGTCTAAATA‐3′.

### 2.12 In vivo mouse tumor model

Mice projects were agreed upon by the Institutional Animal Care and Use Committee of the Fudan and Harbin Medical University according to guidelines and procedures approved. Approved pathogen‐free conditions were maintained to keep athymic nude mice used in this project. All mice were randomly divided into different experimental groups. Note that 2  ×  10^6^ ESCC cells with indicated treatment were introduced into mice flank subcutaneously at the age of 8–9 weeks. Then tumor growth was monitored weekly. Finally, established flank xenografts were removed, and volume, weight and size were calculated. For the experiments with ESCC cells via tail injection, tumors and the lung were collected, formalin‐fixed and photographed after mice were sacrificed. Hematoxylin and eosin and immunohistochemistry staining with Ki67 antibody were performed. In addition, the number of metastatic lung nodules was also calculated.

### 2.13 Purification of GST‐CLK4 protein

Purifying GST‐CLK4 and its mutants in *Escherichia coli* as described previously.^15^

### 2.14 ESCC cell migration, proliferation and colony formation

We carried out transwell assays as reported previously.^16^, ^17^ Briefly, cultured ESCC cells (2 × 10^5^/wells) were resuspended in medium without serum (200 μl) and replated into the 24‐well Boyden chambers’ upper inserts (Corning). Its lower chamber was added medium with 10% FBS, which acts as a chemoattractant. Thirty‐six hours later, paraformaldehyde and crystal violet (.1%) solution were used to fix ESCC cells and observe these migrated cells at room temperature, respectively. The six‐well plate was used to maintain indicated ESCC cells (500/well) for two weeks, used for colony formation analysis. After these cells were treated using 100% methanol, added trypan blue solution for staining and then counted the colonies within a digital camera. The same number of ESCC cells (500/well) was cultured in 96‐well plates. The CCK‐8 kit (Sigma, #96992) was used to evaluate the proliferation of ESCC cells every 24 h. A microplate reader was utilized to count the number of viable cells by recording optical density values at 450 nm. All experiments were conducted at least in triplicate.

### 2.15 Bioinformatics analysis

Bioinformatics about ESCA TCGA RNA sequencing (RNA‐seq) dataset and relevant clinical characteristics of ESCA patients can be available from the websites: https://xenabrowser.net and http://gepia.cancer‐pku.cn/, respectively. We obtained the data about the *CLK4* mRNA array in Gene Expression Omnibus, which can be downloaded on a publicly available website. ESCA patients were divided into three groups (low, intermediate and high) based on the expression levels of the *CLK4* gene. The cutoff value used the 25th and 75th percentiles. According to https://biit.cs.ut.ee/gprofiler/gost, the top 550 genes up‐regulated were available for the analysis of gene ontology. The rates of false discovery were calculated via Benjamini–Hochberg procedure.

### 2.16 Luciferase assay

HEK293T or ESCC cells with .1 μg of *CLK4‐Luc* promoter‐reporter or control reporter were co‐transfected with pRL‐TK Renilla construct (1 ng, Promega). Lipofectamine 2000 reagent (Invitrogen) was used to perform the dual‐luciferase reporter experiments. Two days later, the activity analysis of the luciferase reporter was monitored by using the Dual‐Luciferase Reporter Assay Kit (Promega).

### 2.17 Chromatin immunoprecipitation‐qRT‐PCR

The crosslink of indicated ESCC cells was carried out with formaldehyde (1%) and then terminated by adding .125 M glycine. SDS lysis buffer (200 μl) lysed the indicated ESCC cells. After spinning, the supernatants were mixed with chromatin immunoprecipitation (ChIP) dilution buffer at a ratio of 1:9, and chromatin solutions (2 ml) were precleared with protein G‐Sepharose slurry (Sigma) and incubated with the indicated antibody at 4°C overnight. Immunoprecipitated proteins were recovered with protein G‐Sepharose slurry, and crosslinking of protein‐DNA complexes was performed at 65°C. Then the extracted DNA was used to set up PCR to quantify the signals.

### 2.18 Statistics

Data collected at least in triplicate and reported via mean ± standard deviation (SD) were analysed using GraphPad Prism software 5.0 0 (GraphPad Software Inc.) and SPSS21.0 statistical software (SPSS INC., USA) and R software package (version 3.0.0). The differences comparing two independent groups were confirmed via the Student's *t*‐test. But the differences among more than two groups were assayed with the method of one‐way ANOVA. However, the two‐way ANOVA method was used to elucidate the significance of the growth curves. Correlation between proteins or genes from patient tissues was compared through Pearson correlation analysis. Methylation differences between several groups were clarified with the help of a Mann–Whitney *U* test. The Kaplan–Meier curves and the log‐rank test for the statistical assay were employed. Each biological experiment was performed in triplicate unless otherwise specified. Statistical value * represented *p* < .05, which means a significant difference statistically, whereas **p < .01 or ***p < .001 represented very substantial difference.

## 3 RESULTS

### 3.1 Methylation‐associated down‐regulation of *CLK4* expression is closely related to poor prognosis of aggressive ESCC

**FIGURE 1:**
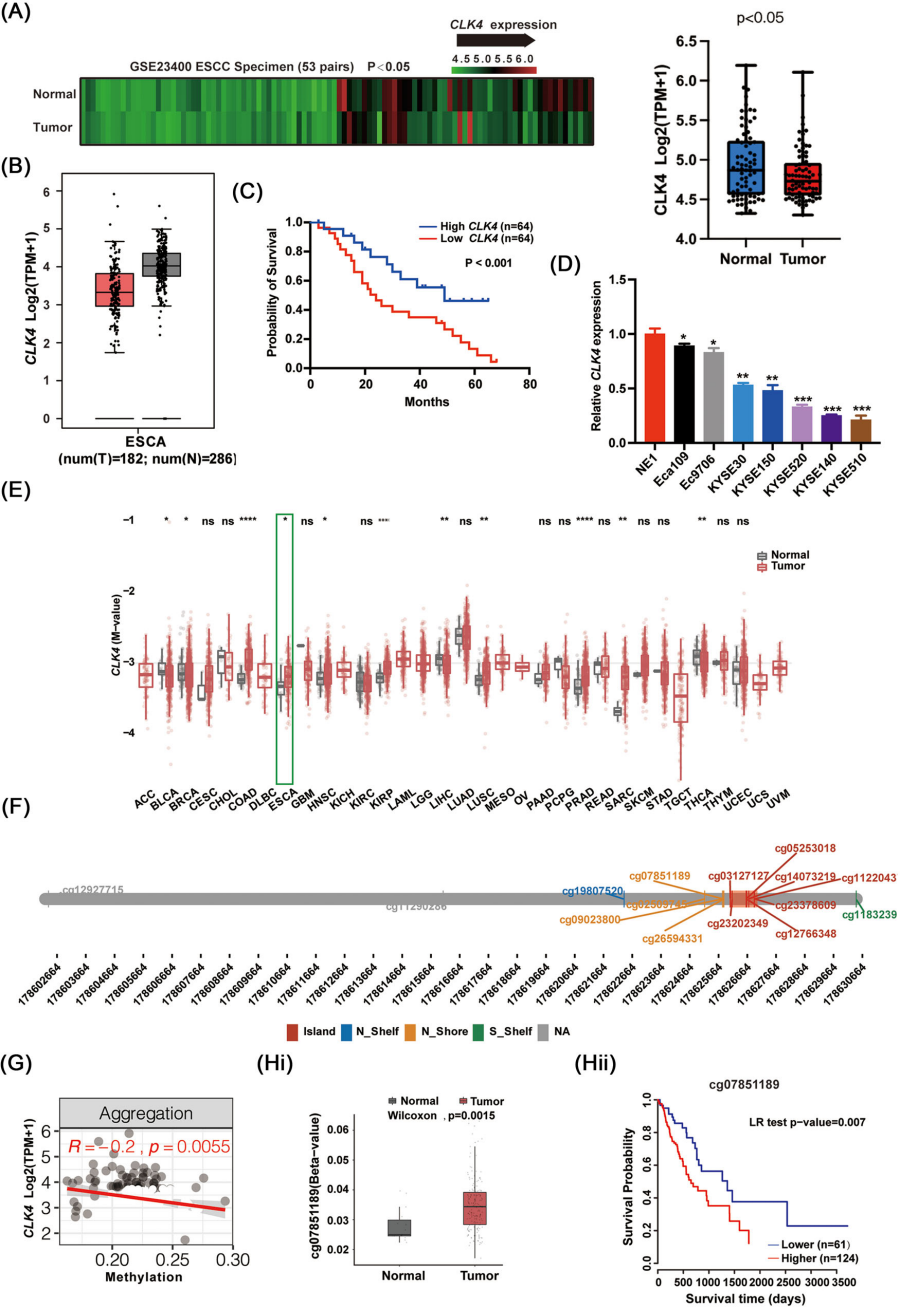
Methylation‐associated down‐regulation of CDC‐like kinase 4 (CLK4) expression explicitly predicts the poor outcomes of aggressive esophageal squamous cell carcinoma (ESCC). (**A**) GSE23400 ESCC database was used to compare the levels of *CLK4* mRNA (*n* = 53). (**B**) The levels of *CLK4* mRNA were compared by using TCGA ESCA data. (**C**) Kaplan–Meier analysis indicated that low expression of *CLK4* predicted poor overall survival. Patients’ samples (*n* = 128) were obtained from Zhongshan Hospital, Fudan University. (**D**) The relative expression of CDC‐like kinase 4 (*CLK4)* mRNA was compared in NE1 cells as control and several ESCC cell lines, which were from three repeated experiments. **(E)** The methylation profiling of *CLK4* promoter in various kinds of tumor tissues and their normal controls. **(F)** Several methylated CpG sites such as cg23378609, cg23202349, cg07851189, cg11220437, cg12766348, cg14073219, cg0312712127. Some of them possibly had a negative correlation with *CLK4* expression in TCGA ESCA (**G**). (**Hi and Hii**) *CLK4* methylation of cg12766348 predicts a poor overall survival (OS) in patients with ESCA

CLK4 expression was first examined in eight paired ESCC patient samples and the controls. As demonstrated in Figure S1A, CLK4 level was lower in ESCC tissues than that in non‐tumor samples. Similar results were observed in an ESCC tissue array containing 120 samples (Figure S1B), implying that CLK4 level is negatively related to the malignant state of ESCC. The above findings were confirmed by assaying the GSE23400 ESCC database (Figure 1A). The TCGA ESCA data in Figure 1B also indicated that compared to those in the normal tissues, *CLK4* levels were reduced in ESCC tumor tissues. And the patients with lower *CLK4* levels had a poorer prognosis (Figure 1C). Consistent with in vivo data, the *CLK4* level in most ESCC cell lines was down‐regulated compared to that in the NE1, a normal oesophageal epithelial cell line (Figure 1D). Therefore, CLK4 might function as a tumor suppressor gene in ESCC.

Given that DNA methylation epigenetically dictated gene expression levels in eukaryotes,^18^ we analysed CpGs residing within *CLK4* using ESCA TCGA samples (Figure S1C). Interestingly, compared to that in the adjacent normal tissues, the *CLK4* promoter was methylated from various kinds of tumors, including ESCA (Figure 1E). By analysing methylation beadchip from TCGA Illumina 450 k Infinium, at least six CpG methylated sites were observed, such as cg23378609, cg23202349, cg07851189, cg12766348, cg14073219 and cg0312712127 (Figure 1F). Some of them possibly had a negative correlation with CLK4 expression (Figure 1G). Significantly, the methylation in cg07851189 CpG predicted a poor prognosis in ESCA patients compared to the hypomethylation (Figure 1Hi,ii).

To exclude the causality between methylation of the given CpG sites and the expression of CLK4, we treated ESCC cells with 5‐aza‐2′‐deoxycytidine (5′‐Aza), a DNA methyltransferase (DNMT) inhibitor. As shown in Figure S1D, the levels of CLK4 mRNA were increased after 5′‐Aza treatment compared with the control. We also observed histone methylation such as H3K9me3 and H3K27me3 in the CLK4 promoter region, as shown in Figure S1E. Figure S1F indicated that in the other nine CpG sites, three sites (cg26594331, cg02509745 and cg12927715) were methylated; cg19807520 was hypomethylated, and no methylation was observed for the other four CpG sites.

Together, methylation‐associated down‐regulation of CLK4 expression may predict the poor outcomes of aggressive ESCC.

### 3.2 CLK4 down‐regulation contributes to the malignant state of ESCC

**FIGURE 2:**
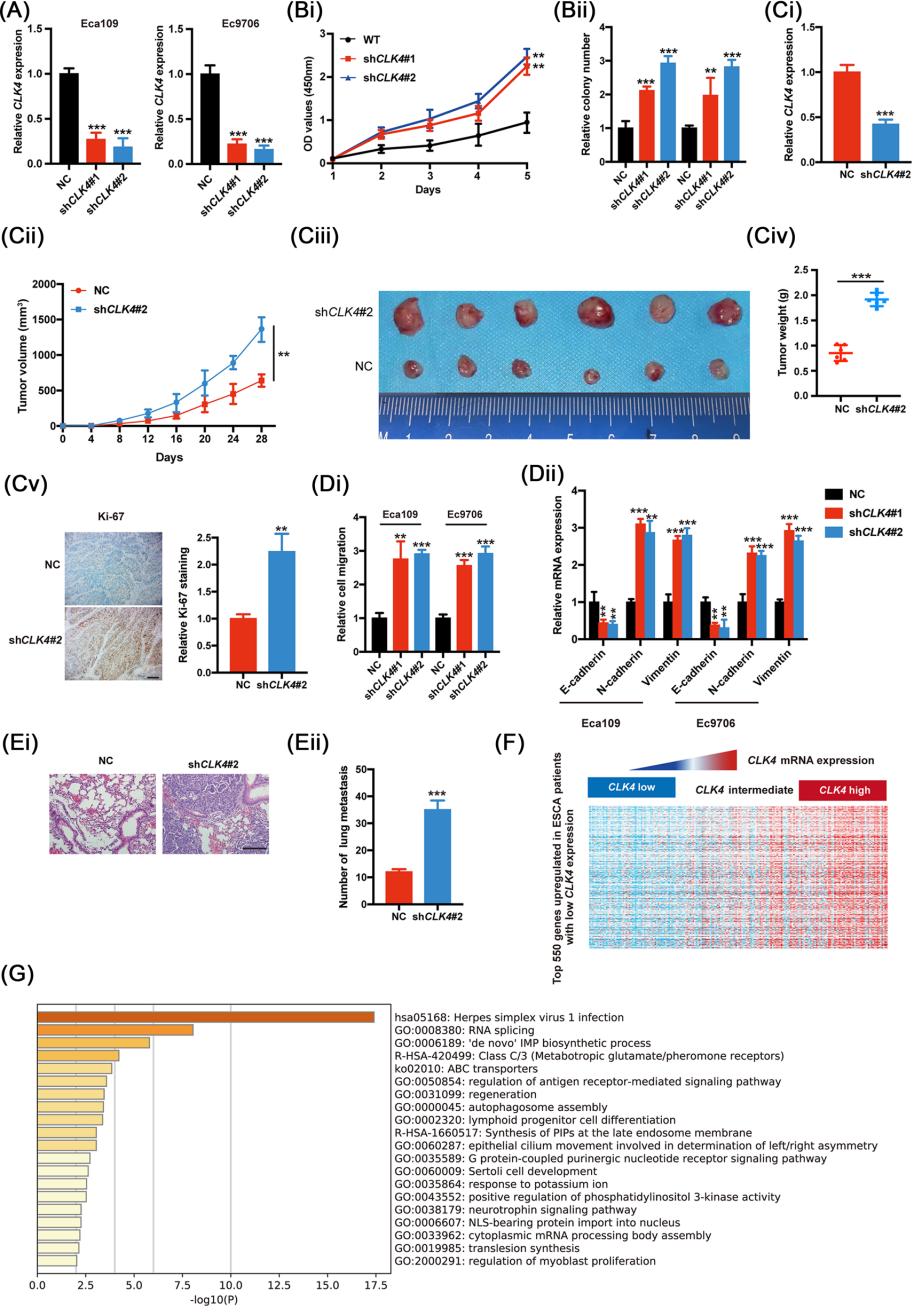
CDC‐like kinase 4 (CLK4) down‐regulation contributes to the malignant state of esophageal squamous cell carcinoma (ESCC). **(A)** qRT‐PCR of *CLK4* confirmed the efficiency of shRNA#1 and #2 in two indicated ESCC cell lines. (**Bi**) The growth curves were performed using the CCK8 kit in indicated cells as in (**A**). (**Bii**) Colony formation was performed in indicated cells as in (**A**). Data in **(Bi)** and (**Bii)** indicate the mean ± SD, which were from three repeated experiments. (**Ci**) The levels of *CLK4* mRNA were measured in the tissues of dissected tumors using qRT‐PCR. (**Cii**) The volumes of the tumor (*n* = 6 in each group) were shown in a time‐course manner. And *CLK4* in Eca109 cells was stably knocked down. (**Ciii**) The representative pictures of the xenografted tumors as indicated. (**Civ**) Tumor weight was shown as indicated. (**Cv**) The effects of *CLK4* knockdown on tumor Ki67 staining. Representative images and statistical quantification were presented. Knocking down *CLK4* affected the migrative ability (**Di**) and epithelial‐mesenchymal transition (EMT)‐related genes’ levels (**Dii**) of Eca109 and Ec9706, which were from three repeated experiments. (**Ei**) Hematoxylin and eosin (H&E) staining images indicated that knocking down *CLK4* affected lung metastasis of ESCC (*n* = 6/group). (**Eii**) The nodule number of lung metastasis was shown. (**F**) Heat map indicated that *CLK4* levels affect the transcriptomics in ESCC patients. (**G**) The top 20 function pathways affected by *CLK4* levels in ESCC patients were enriched and presented

To elucidate the performance of CLK4 in ESCC, we first down‐regulated CLK4 expression in the Eca109 and Ec9706 cell lines (Figure 2A), which leads to enhanced proliferation and colony formation capability of ESCC cell lines (Figure 2Bi,ii). These in vitro findings were confirmed using a xenografted nude model in an in vivo experiment (Figure 2Ci‐v).

Moreover, migration capacity and EMT‐related genes’ levels were upregulated once *CLK4* was knocked down in Eca109 and Ec9706 cells (Figure 2Di,ii). In the metastasis models, similar results were observed in the *CLK4*‐knockdown group (Figure 2Ei,ii).

Conversely, CLK4 overexpression in KYSE510 and KYSE140 cell lines had the opposite effects on ESCC (Figure S2).

To clarify the mechanisms by which *CLK4* acts, the ESCA TCGA database was divided into *CLK4* ^high^ and *CLK4* ^low^ groups (Figure 2F). And function enrichment of the top 550 genes in either group implies that *CLK4* level is possibly related to tumor metabolism reprogramming (the third enriched pathway) (Figure 2G and Table S1).

Together, CLK4 down‐regulation promotes malignancy of ESCC cells possibly via metabolic pathway reprogramming.

### 3.3 Knockdown of CLK4 defines a novel oncogenic pathway by reprogramming purine metabolism and redox status in ESCC

**FIGURE 3:**
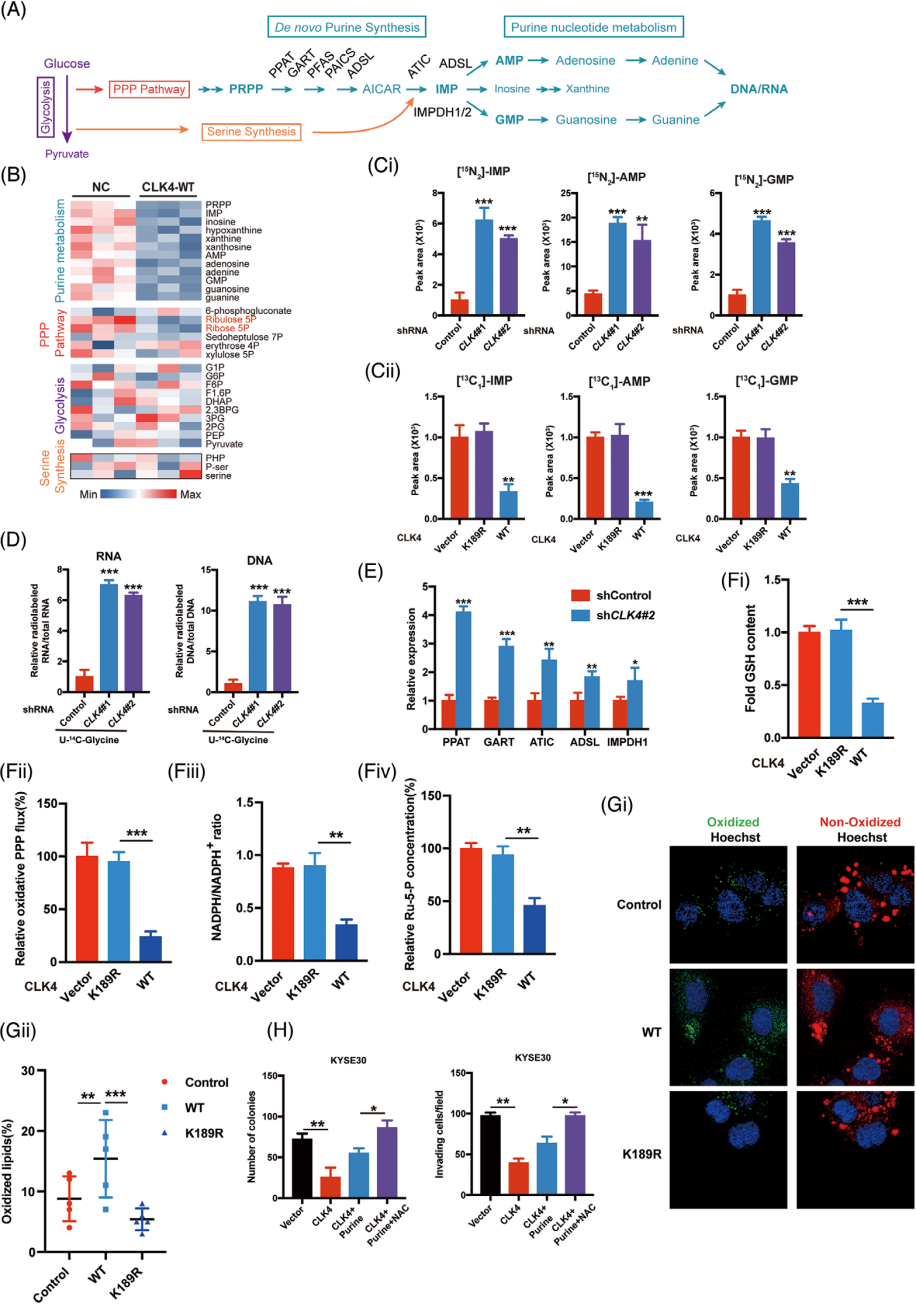
Knockdown of CDC‐like kinase 4 (CLK4) defines a novel oncogenic pathway by reprogramming purine metabolism and redox status in esophageal squamous cell carcinoma (ESCC). (**A**) Schematic diagram of de novo purine synthesis pathway. (**B**) Heat map showing the effects of CLK4 on the metabolites of KYSE140 cells, which were examined by liquid chromatography with tandem mass spectrometry (LC‐MS/MS). (**Ci**) Stable isotope‐labelled glutamine (amide‐^15^N) was used to examine the effects of *CLK4* knockdown on inosine monophosphate (IMP), guanosine monophosphate (GMP) and adenosine monophosphate (AMP) synthesis in Eca109 cells. (**Cii**) Stable isotope‐labelled ^13^C‐glycine was used to examine the effects of WT‐CLK4 or its K189R mutant on IMP, GMP, and AMP synthesis in Eca109 cells. (**D**) Radio‐labelled ^14^C‐glycine was used to examine the effects of *CLK4* knockdown on the levels of RNA and DNA in Eca109 cells. (**E**) The effects of *CLK4* knockdown in Eca109 cells on the purine synthesis‐related enzymes. (**Fi‐iv** and **G**) The effects of WT‐CLK4 and CLK4‐K189R on GSH, oxidative PPP flux, NADPH/NADP^+^ ratio, ribulose‐5‐phosphate (Ru‐5‐P), and lipid peroxidation. (**H**) Purine and N‐Acetyl‐L‐cysteine (NAC) supplementation reverted the effects of CLK4 overexpression on ESCC malignant state. The above results were from three repeated experiments

We then performed metabolic profiling of ESCC cells to elucidate the effect of CLK4 on ESCC‐related metabolic pathways via MS.^16^ Interestingly, CLK4 up‐regulation did not significantly change glycolysis and serine synthesis, while Ru‐5‐P from the PPP pathway and purine‐related intermediates were down‐regulated (Figure 3A,B; and Table S2). Therefore, we hypothesized that the purine synthesis pathway and NADPH levels might be up‐regulated in *CLK4*‐knockdown ESCC cells.

To confirm this hypothesis, we carried out the experiments of stable isotope‐labelled glutamine or glycine. As shown in Figure 3C, knocking down *CLK4* enhanced ^15^N‐purine intermediates, whereas *WT‐CLK4* but not inactive *CLK4‐K189R* mutant decreased IMP, AMP, and guanosine monophosphate (GMP) in ESCC cells. Enhanced ^14^C‐glycine flux into DNA and RNA and purine‐related enzymes were also observed in ESCC cells with *CLK4* knockdown (Figure 3D,E). Moreover, *WT‐CLK4* but not *CLK4‐K189R* mutant in ESCC cells inhibited GSH, oxidative PPP flux, NADPH/NADP^+^ ratio and ribulose‐5‐phosphate (Ru‐5‐P) but enhanced lipid peroxidation (Figure 3F,G). Furthermore, purine and NAC supplementation rescued *CLK4*‐mediated inhibition of ESCC cells (Figure 3H), indicating that *CLK4* knockdown contributes to ESCC development possibly through promoting purine synthesis and deoxidation.

### 3.4 MITF is identified as a critical effector downstream of CLK4 in ESCC

**FIGURE 4:**
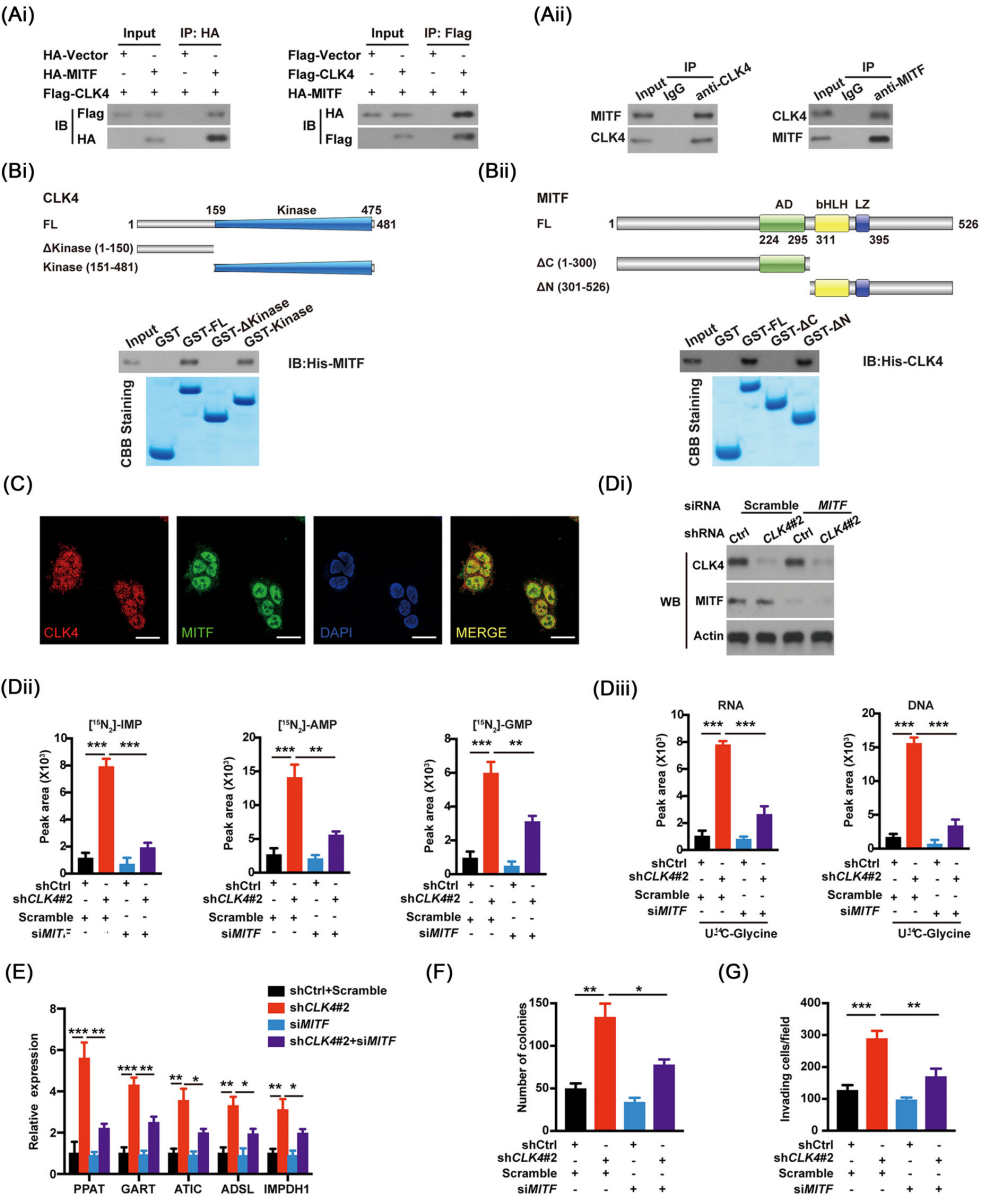
Microphthalmia‐associated transcription factor (MITF) is identified as a critical effector downstream of CDC‐like kinase 4 (CLK4) in esophageal squamous cell carcinoma (ESCC). **(Ai)** HEK293T cells were infected with indicated constructs. Then co‐immunoprecipitation (co‐IP) and western blots were carried out using indicated antibodies. (**Aii**) Endogenous co‐IP between CLK4 and MITF was performed in Eca109 cells using antibodies as indicated. (**Bi**) Analysis of the domains of human CLK4 necessary for the interaction with MITF. (**Bii**) Analysis of the domains of human MITF necessary for the interaction with CLK4. Western blots and Coomassie brilliant blue (CBB) staining were shown. (**C**) Representative images of cellular localization for CLK4 and MITF in HEK293T cells. (**Di**) The confirmation of *MITF* and *CLK4* knockdown in Eca109 cells. (**Dii**) *MITF* knockdown reverted the effects of *CLK4* knockdown on IMP, AMP and GMP in Eca109 cells. (**Diii**) *MITF* knockdown reverted the effects of CLK4 knockdown on RNA and DNA in Eca109 cells. (**E**) Knocking down *MITF* reverted the expression of purine‐related enzymes in Eca109 cells with *CLK4* knockdown. (**F‐H**) Knocking down *MITF* reverted the NADPH/NADP^+^ ratio (**F**), capability of colony formation (**G**) and invasion (**H**) of Eca109 cells with *CLK4* knockdown. The above results were from three repeated experiments

To clarify the mechanisms by which CLK4 reprograms redox homeostasis and purine metabolism in ESCC, we performed the proximity‐dependent biotin (BioID2) assays with ESCC cells. The results identified MITF as a bona fide interactor of CLK4 (Figure S3A). Given that MITF has been shown as an important TF in regulating redox signaling and nucleotide metabolism,^19^ we reasoned that MITF might mediate the effect of *CLK4* knockdown on the ESCC. To test this idea, we first confirmed the interaction between MITF and CLK4 using endogenous and exogenous co‐IP (Figure 4Ai,ii). Notably, the kinase domain of CLK4 was necessary for its interaction with MITF, whereas the C‐terminus of GST‐MITF was required for its binding to CLK4 (Figure 4Bi,ii). Both CLK4 and MITF were observed primarily in the nuclear localization of HEK293T cells (Figure 4C), suggesting the possible involvement of MITF in the role of CLK4. As expected, co‐transfection of *MITF* reverted the decrease in purine synthesis in *CLK4*‐up‐regulated ESCC cells (Figure S3B). On the contrary, *MITF* knockdown rescued the effects of *CLK4* knockdown on purine metabolism in ESCC cells (Figure 4 Di‐iii,E). Also, *MITF* knockdown rescued the effects of *CLK4* knockdown on the NADPH/NADP^+^ ratio (Figure 4F). *MITF* knockdown also rescued the effects of *CLK4* knockdown on the malignant state of ESCC (Figure 4G,H).

Therefore, MITF is a vital effector downstream of CLK4 in ESCC.

### 3.5 CLK4 phosphorylates MITF at Y360

**FIGURE 5:**
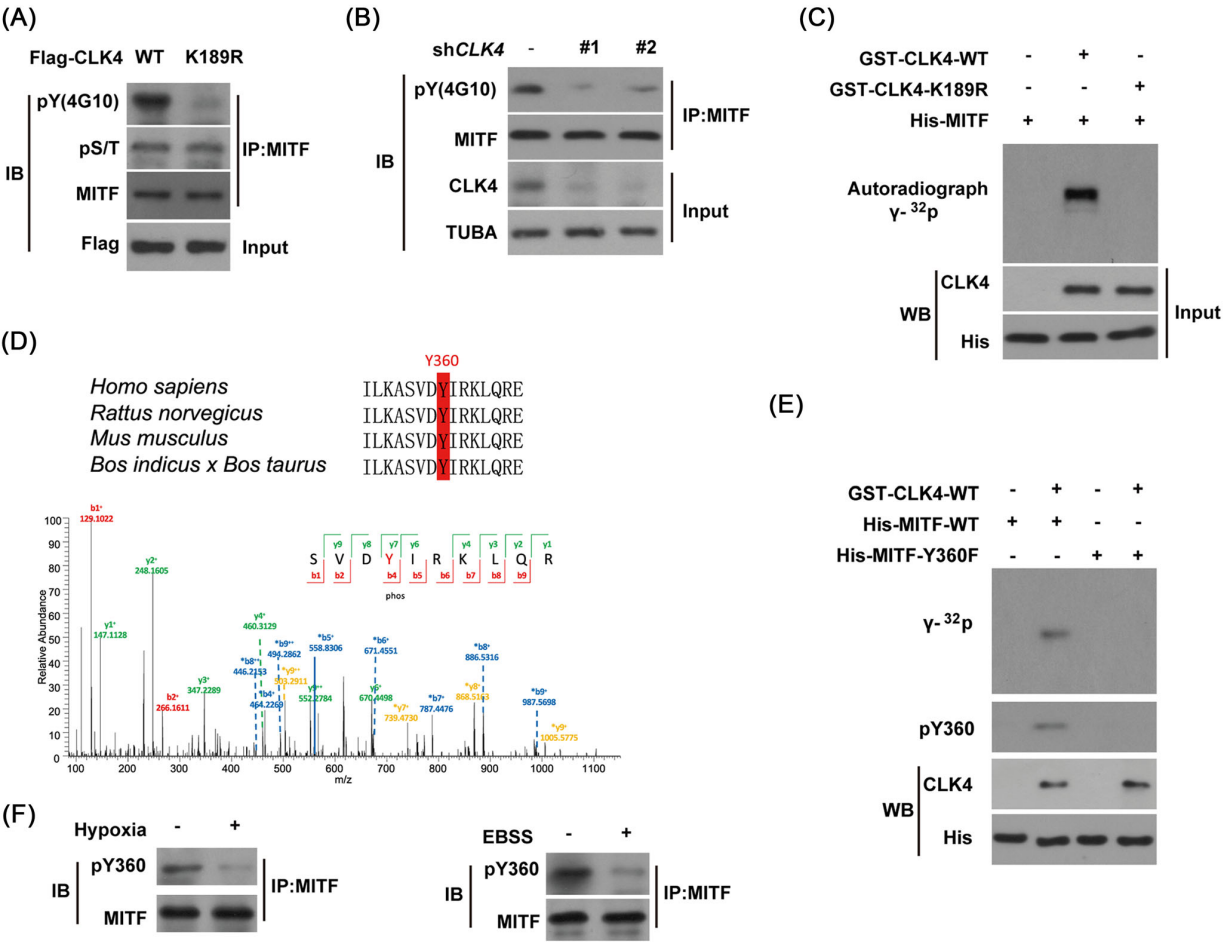
CDC‐like kinase 4 (CLK4) phosphorylates microphthalmia‐associated transcription factor (MITF) at Y360. **(A)** KYSE510 cell lines were transfected with *WT‐Flag‐CLK4* or kinase‐deficient mutant *Flag‐CLK4‐K189R*. Then immunoprecipitated MITF was probed with anti–phosphor‐ser/thr antibody and anti‐tyrosine 4G10 antibody, respectively. (**B**) *CLK4* was knocked down in the Eca109 cell line, and the tyrosine phosphorylation of MITF was examined using an anti‐4G10 antibody. (**C**) An in vitro phosphorylation assay was established to observe whether purified glutathione S‐transferase (GST)‐CLK4‐WT or K189R mutant phosphorylated His‐MITF. (**D**) The conserved Y360 was identified as MITF phosphorylation residue by CLK4 with mass spectrometry. (**E**) A specific anti‐phospho‐MITF Y360 antibody could not recognize MITF‐Y360F mutation. (**F**) Hypoxia and Earle's balanced salt solution (EBSS) (a phosphate buffer used to simulate cell starvation) treatment increased MITF Y360 phosphorylation in KYSE510 cells. The above results were from three repeated experiments

Since CLK4 is a kinase, and its kinase domain is required for the binding of MITF (Figure 4B), we then examined whether MITF was a substrate of CLK4. To test this idea, the endogenous MITF in KYSE510 cells with WT‐CLK4 or kinase‐deficient mutant CLK4‐K189R was immunoprecipitated, and its phosphorylation was detected with anti–phosphor‐ser/thr antibody (pS/T) or anti‐tyrosine 4G10 antibody, respectively. The results indicated that only 4G10 levels of MITF were up‐regulated in the KYSE510‐CLK4‐WT not K189R mutant cells and that there was no noticeable difference in the phosp‐ser/thr levels (Figure 5A). Conversely, the tyrosine phosphorylation of MITF was impaired in Eca109 cells with CLK4 knockdown but not in control cells (Figure 5B).

Furthermore, we demonstrated that GST‐CLK4‐WT, not K189R mutant phosphorylated His‐MITF at conserved Y360 (Figure 5C,D). And WT‐MITF rather than MITF‐Y360 F mutation was recognized by an anti‐phospho‐MITF‐Y360 antibody (pY360, Figure 5E). Next, we tried to elucidate the pathophysiological conditions, which can induce this phosphorylation. It has been reported that hypoxia induces MITF transcriptional repression in melanoma.^20^ Additionally, Figure 2G suggests a possible relevance of CLK4 with the autophagic pathway. Therefore, we tried to investigate whether hypoxia and Earle's balanced salt solution (EBSS) treatment could induce MITF Y360 phosphorylation in ESCC cell lines. Unsurprisingly, hypoxia or EBSS treatment increased MITF Y360 phosphorylation in ESCC cells (Figure 5F). Given that both hypoxia and serum starvation promote autophagy, we reasoned that autophagy might affect MITF levels in ESCC with dysregulated CLK4.

### 3.6 Phosphorylation of MITF by CLK4 promotes its autophagy‐dependent degradation

**FIGURE 6:**
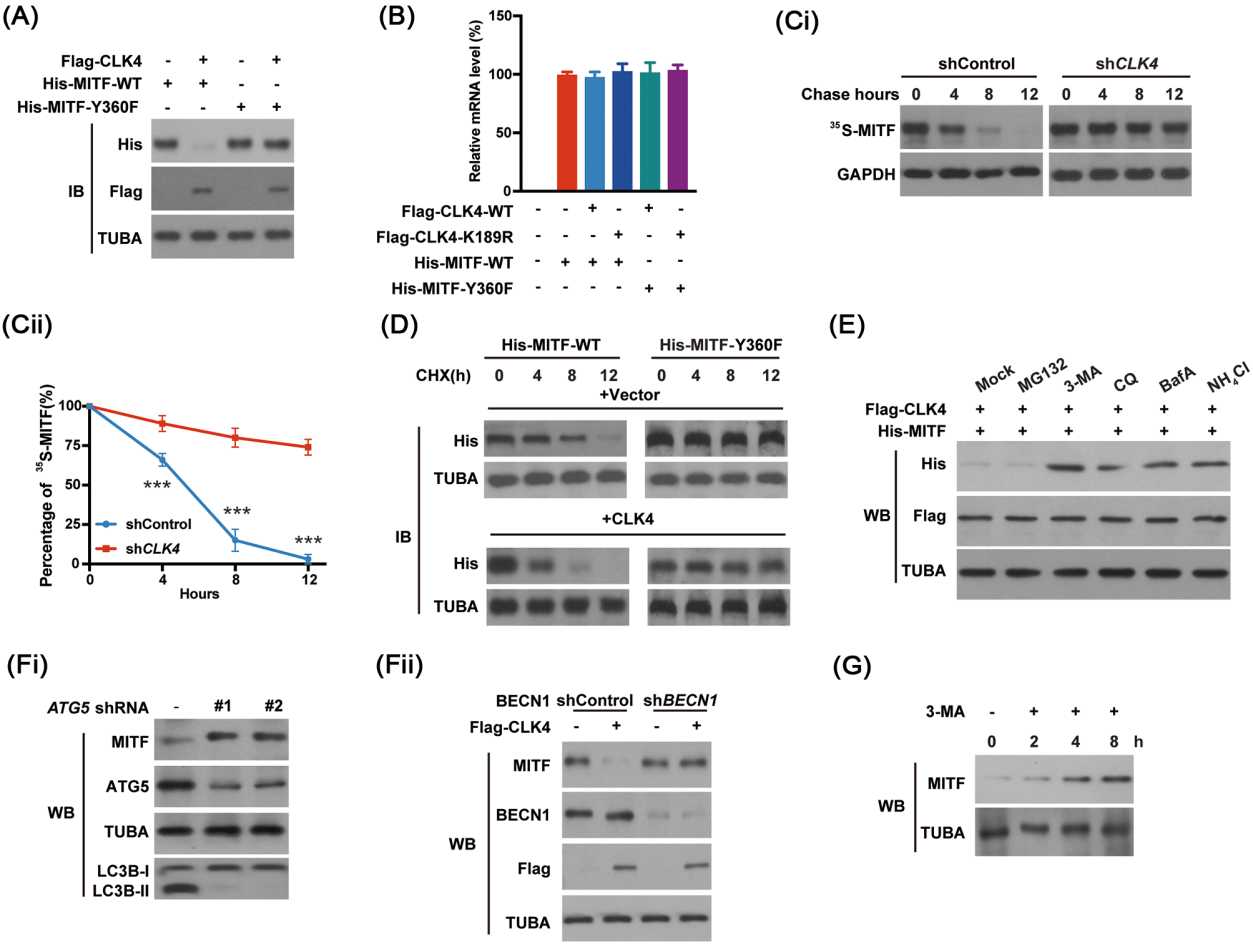
Phosphorylation of microphthalmia‐associated transcription factor (MITF) by CDC‐like kinase 4 (CLK4) promotes its autophagy‐dependent degradation. (**A**) *WT‐His‐MITF* or *His‐MITF‐Y360F* was transfected with or without *Flag‐CLK4* into HEK293T cells. Then immunoblots were carried out using antibodies as marked. (**B**) The effects of WT‐CLK4or CLK4‐K189R on the levels of *MITF* mRNA were examined in HEK293T using qRT‐PCR. (**Ci‐ii**) MITF levels were monitored in metabolically labelled HEK293T cells with [^35^S]‐methionine. (**D**) Cycloheximide (CHX) assay was used to observe the stability of MITF in HEK293T cells treated as indicated. (**E**) HEK293T cells infected with indicated constructs were treated with MG132 (10 μM), 3‐MA (10 mM), chloroquine (CQ) (50 μM), Baf A (.2 μM), and NH_4_Cl (20 mM) for 8 h, respectively. Immunoblots were performed as indicated. (**Fi‐ii**) *ATG5* or *BECN1* shRNA was transfected into HEK293T cells along with or without *Flag‐CLK4*; the levels of MITF were examined. (**G**) 3‐MA (2 mM) treatment increased MITF accumulation in KYSE510 cells. The above results were from three repeated experiments

Protein phosphorylation is usually linked to its stability.^21^ It was noticed that overexpression of *CLK4* in HEK293T cells down‐regulated the levels of WT‐MITF rather than MITF‐Y360F (Figure 6A). However, *CLK4* overexpression did not change the mRNA levels of *MITF* (Figure 6B). Consistently, *CLK4* knockdown in HEK293T significantly prolonged the half‐life of MITF compared with the control group (Figure 6Ci,ii). And MITF‐Y360F, not WT‐MITF, was more resistant to degradation induced by CLK4 (Figure 6D). Therefore, we conclude that phosphorylation of MITF at Y360 disrupts protein stability.

The lysosome, or proteasome, or autophagic pathway controls protein degradation.^22^ Next, we wanted to elucidate how phosphorylation of MITF by CLK4 at Y360 reduces its stability. As shown in Figure 6E, CLK4‐mediated degradation of MITF could be prevented by 3‐methyladenine (3‐MA, an autophagy inhibitor) or autolysosome inhibitors such as NH_4_Cl, bafilomycin A1 (BafA), and chloroquine. However, MG132 (a proteasome inhibitor) did not block MITF degradation (Figure 6E). In addition, in *ATG5* or *BECN1* knockdown cells, we did not observe CLK4‐triggered degradation of MITF (Figure 6Fi,ii). Consistently, the addition of 3‐MA impeded the degradation of MITF (Figure 6H). Together, these results suggest that CLK4 promotes autophagic degradation of MITF in ESCC.

### 3.7 CLK4 enhances the interaction between MITF and SQSTM1, which mediates autophagic degradation of MITF

**FIGURE 7:**
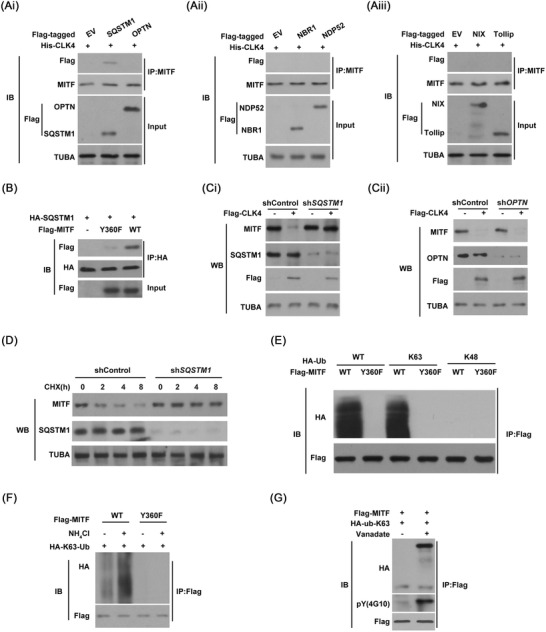
CDC‐like kinase 4 (CLK4) enhances the interaction between microphthalmia‐associated transcription factor (MITF) and SQSTM1, which mediates autophagic degradation of MITF. (**Ai‐iii**) Autophagic receptors with *His‐CLK4* were co‐introduced into HEK293T cells, respectively. Then MITF was immunoprecipitated, and its binding to these receptors was probed. (**B**) HEK293T cells were transfected, and co‐immunoprecipitation (co‐IP) was performed as shown. (**Ci** and **Cii**) In WT and SQSTM1 or optineurin (OPTN) knockdown HEK293T cells with *Flag‐CLK4*, MITF levels were examined using immunoblots. (**D**) In WT and SQSTM1 knockdown HEK293T cells, cycloheximide (CHX) assays were performed as indicated. **(E)** Y360F mutation decreased the K63‐poly‐Ub binding of MITF in HEK293T cells. **(F)** NH_4_Cl (15 mM) treatment promoted the ubiquitination of MITF in KYSE510 cells. **(G)** Vanadate (100 nM) treatment promoted K63 poly‐ubiquitination of MITF in KYSE510 cells. The above results were from three repeated experiments

Autophagic receptors (such as SQSTM1, Tollip, NDP52, OPTN, Nix and NBR1 [NBR1 autophagy cargo receptor]) are shown to be necessary for selective degradation.^23^ Next, we tried to identify the autophagic receptor essential for MITF degradation. We found that CLK4 promoted the association of MITF with SQSTM1 rather than other autophagic receptors (Figure 7Ai‐iii). However, MITF‐Y360F did not bind to SQSTM1 (Figure 7B). We also observed that CLK4 failed to lead to a reduction in MITF protein levels in *SQSTM1* knockdown rather than *OPTN* knockdown cells (Figure 7Ci,ii). Moreover, *SQSTM1* knockdown impaired MITF degradation in cells (Figure 7D). Collectively, our data suggest that SQSTM1 is the autophagic receptor for MITF selective degradation.

Since substrate ubiquitination was required for the recognition of autophagic receptor SQSTM1,^24^ we reasoned that CLK4‐mediated MITF phosphorylation might lead to its ubiquitination. Indeed, WT‐MITF rather than MITF‐Y360F mutant was observed to bind more K63‐only but not K48‐only ubiquitin (Figure 7E). NH_4_Cl treatment enhanced the ubiquitination of WT‐MITF rather than Y360F mutant (Figure 7F). In addition, vanadate, a general inhibitor for phosphotyrosyl phosphatases, up‐regulated the tyrosine phosphorylation and K63 poly‐ubiquitination of MITF (Figure 7G). Together, our findings support the model in which CLK4‐mediated tyrosine phosphorylation promotes selective degradation of MITF via the K63‐ubiquitin.

### 3.8 E3 ligase COP1 is required for MITF K63‐linked ubiquitination

**FIGURE 8:**
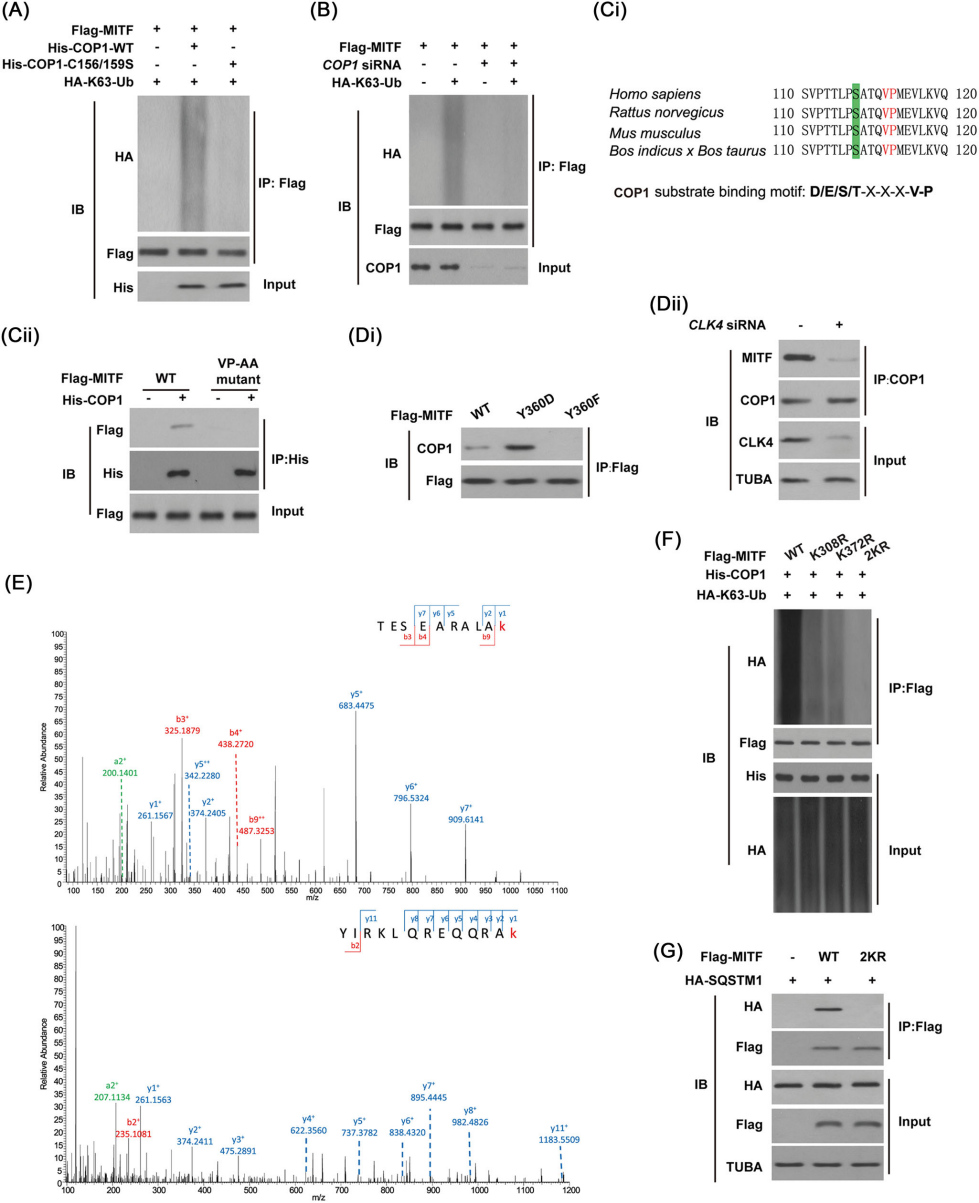
E3 ligase COP1 is required for microphthalmia‐associated transcription factor (MITF) K63‐linked ubiquitination. (**A and B**) The effects of *WT‐COP1* or its inactive mutant or its knockdown on the ubiquitination of MITF in HEK293T cells. (**Ci**) A conserved VP motif was highlighted in sequences of MITF, and it conformed to the COP1 binding motif reported previously. (**Cii**) The VP motif was necessary for the interaction between MITF and COP1 in HEK293T cells. (**Di**) Co‐immunoprecipitation (co‐IP) was carried out in indicated esophageal squamous cell carcinoma (ESCC) cells. (**Dii**) CDC‐like kinase 4 (*CLK4)* siRNA or scramble was performed in ESCC cells, and co‐immunoprecipitation (co‐IP) was carried out using indicated antibodies. (**E**) Mass spectrometry (MS/MS) identified two possible MITF K63‐linked ubiquitination sites by COP1. (**F**) In vivo ubiquitination of MITF was analysed in ESCC cells. (**G**) *WT‐MITF* and its *K308/372R* mutant were transfected into ESCC cells, and the immunoblots were performed with indicated antibodies. The above results were from three repeated experiments

Next, we tried to screen the E3 ligase responsible for the K63‐linked poly‐ubiquitination of MITF. Our MS/MS data identified 5 E3 ligases (Fbxo7, KLHL20, Trim25, Parkin and COP1) as possible interactors of MITF. However, only co‐transfection of COP1 rather than the other 4 ligases markedly promoted K63‐linked ubiquitination of MITF (Figure 8A). And COP1‐C156/159S, an E3 ligase‐inactive mutant,^25^ could not induce MITF ubiquitination (Figure 8A). COP1 deficiency had the opposite effects (Figure 8B).

The VP motif preferentially mediates the interaction between COP1 to its target proteins.^26^ Examining the amino acid sequence of MITF protein identified a putative motif of COP1 binding, located in MITF (aa 110–120) (Figure 8Ci). It was found that the mutant of the VP motif to alanine (VP→AA) disrupted the association of COP1 with MITF (Figure 8Cii), indicating that one direct interaction between MITF and COP1 is critical for MITF K63‐lined ubiquitination.

Co‐IP indicated that CLK4‐mediated MITF phosphorylation at Y360 promoted its association with COP1 (Figure 8Di). Conversely, knockdown of *CLK4* decreased the binding of MITF to COP1 (Figure 8 Dii), indicating that CLK4‐mediated MITF phosphorylation at Y360 is important for the interaction between MITF and COP1.

Furthermore, MS/MS identified K308 and K372 of MITF as two possible ubiquitination sites by COP1 (Figure 8E), confirmed by in vivo ubiquitination assays (Figure 8F). Notably, MITF‐K308/372R mutant could not bind to SQSTM1 (Figure 8G), consistent with the previous data. Therefore, we conclude that COP1 is the E3 ubiquitin ligase for the K63‐linked poly‐ubiquitination of MITF.

### 3.9 ZRANB1 rather than USP13 antagonizes COP1‐mediated MITF ubiquitination

**FIGURE 9:**
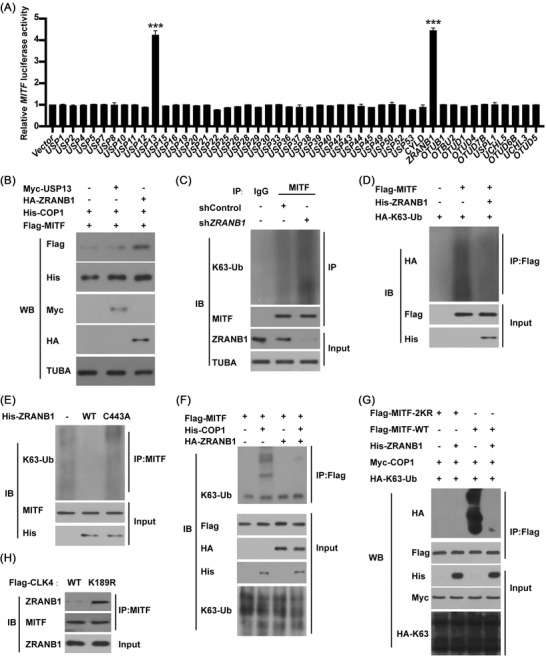
ZRANB1 not USP13 antagonizes COP1‐mediated microphthalmia‐associated transcription factor (MITF) ubiquitination. (**A**) A *MITF*‐Luc firefly reporter was used to identify the putative deubiquitinase (DUBs) of MITF in HEK293T cells. (**B**) Western blots were performed in HEK293T cells treated with constructs as indicated. (**C**) The effects of ZRANB1 knockdown on the ubiquitination of MITF in esophageal squamous cell carcinoma (ESCC) cells. (**D**) The effects of ZRANB1 on the ubiquitination of MITF in HEK293T cells treated as indicated. (**E**) The effects of exogenous ZRANB1 or its inactive mutant (C443A) on the ubiquitination of MITF in HEK293T cells. (**F**) The effects of ZRANB1 on the ubiquitination of MITF in HEK293T cells transfected with or without exogenous COP1. (**G**) The effects of MITF‐K308/372R (2KR) mutant on the ubiquitination of MITF in HEK293T cells treated as indicated. (**H**) The effects of CDC‐like kinase 4 (CLK4) on the binding of ZRANB1 to MITF in HEK293T cells treated as indicated. The above results were from three repeated experiments

Next, a fusion reporter (MITF‐Luc) was constructed and used to search for a putative deubiquitinase (DUB) that might rescue COP1‐mediated MITF ubiquitination. Figure 9A indicated that both ZRANB1 and USP13^27^, ^28^ might mediate the deubiquitination of MITF.

Although USP13 was previously shown to deubiquitinate MITF,^28^ our data indicated that ZRANB1 rather than USP13 enhanced the abundance of MITF protein and inhibited COP1‐mediated MITF degradation (Figure 9B). Conversely, *ZRANB1* knockdown increased the K63‐linked ubiquitination of MITF (Figure 9C). However, WT‐ZRANB1 rather than inactive mutants (C443A) inhibited the K63‐linked ubiquitination of MITF^13^ (Figure 9D–F). And ZRANB1 did not affect MITF‐K308/372R mutant ubiquitination (Figure 9G). Significantly, WT‐CLK4 disrupted the interaction between ZRANB1 and MITF (Figure 9H). Together, these findings indicate that ZRANB1 affects COP1‐mediated ubiquitination and stability of MITF as a DUB.

### 3.10 MITF directly binds and transcriptionally inhibits the promoter of *CLK4* in ESCC

**FIGURE 10:**
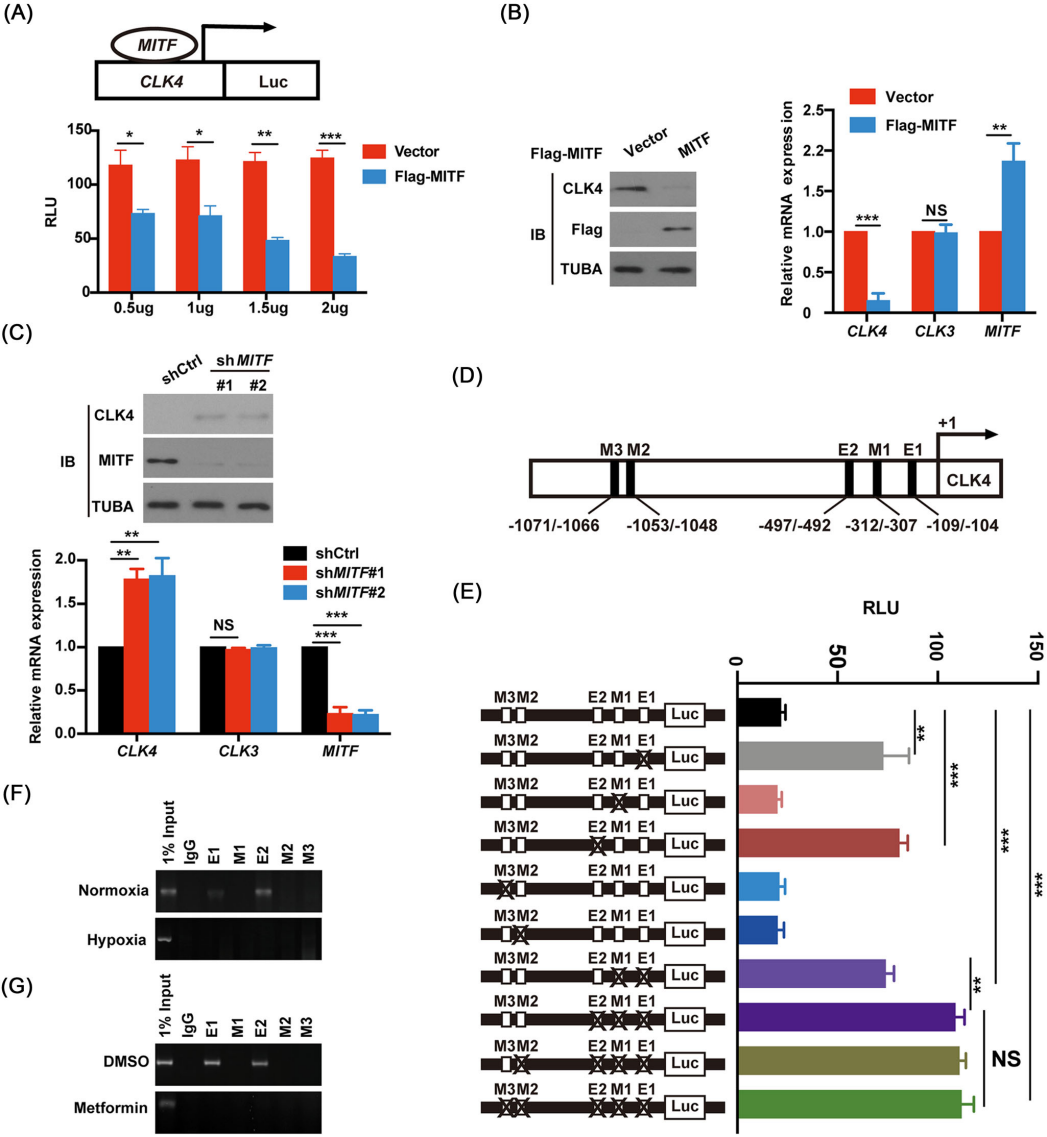
Microphthalmia‐associated transcription factor (MITF) directly binds and transcriptionally inhibits the promoter of CDC‐like kinase 4 (*CLK4)* in esophageal squamous cell carcinoma (ESCC). (**A**) A *CLK4‐Luc* (−1200 bp from transcription start site) luciferase reporter was co‐transfected with or without increasing amounts of *Flag‐MITF* in HEK293T cells. Then the relative luciferase activity (RLU) was examined, and the levels of cytomegalovirus (CMV)‐LacZ were used to normalize the data. (**B**) The indicated plasmids were introduced into ESCC cells. qRT‐PCR and western blots with indicated antibodies were carried out. (**C**) Short Hairpin RNA (ShRNA) targeting against *MITF* (100 nmol/L each) for 48 h. qRT‐PCR and western blots with indicated antibodies were carried out. (**D**) M‐box motif (5′‐CAT/CGTG‐3′) and E‐box motif (5′‐CANNTG‐3′) in the promoter of *CLK4* were shown. (**E**) The effects of E‐ and M‐box mutants on the activity of *MITF‐Luc* reporter were examined in HEK293T cells. (**F**) The effects of hypoxia on the binding of MITF to E‐boxes and M‐boxes in the promoter of *MITF* by ChIP assays. (**G**) The effects of Metformin (10 mM) and dimethyl sulfoxide (DMSO) treatment on the binding of MITF to E‐boxes and M‐boxes in the promoter of *MITF* by ChIP assays. The above results were from three repeated experiments

MITF is one of the transcription factor (bHLH)‐leucine zipper MITF/TFE family TFs.^28^ We then constructed a *CLK4*‐Luc luciferase reporter of the human *CLK4* promoter and examined whether MITF transcriptionally affected the activity of *CLK4*‐Luc. As shown in Figure 10A, MITF dose‐dependently inhibited the activity of *CLK4*‐Luc. Moreover, immunoblot and qRT‐PCR assays showed that overexpression of *MITF* suppressed endogenous *CLK4* rather than another CLK family member *CLK3* mRNA expression in ESCC cells (Figure 10B). However, knocking down *MITF* had the opposite effect (Figure 10C).

Since MITF regulates gene expression by binding to specific motifs known as M‐box and E‐box,^28^ the promoter sequence of *CLK4* was examined, which reveals two E‐box motifs and three M‐box sites (Figure 10D). It was demonstrated that E‐box rather than M‐box mutation from CAxxTG to ATxxTA significantly up‐regulated the level of *CLK4* (Figure 10E), suggesting that MITF inhibits CLK4 expression by binding to E‐boxes 1 and 2. Interestingly, hypoxia induced the binding of *MITF* to two E‐box motifs in the promoter of *CLK4* in ESCC cells (Figure 10F). Because Metformin was known to repress ESCC progression,^29^ we examined whether Metformin regulated *MITF* binding to the promoter of *CLK4* in ESCC cells. ChIP‐PCR assays indicated that the binding of *MITF* to two E‐boxes in the *CLK4* promoter was abolished in ESCC cells (Figure 10G).

We also transfected HEK293T cells and ESCC cells with Flag‐MITF‐Y360F mutant, or Flag‐MITF‐Y360D phosphorylation mimic mutant with a luciferase reporter of *CLK4*‐luc, respectively. The results indicated that MITF‐Y360F mutant rather than MITF‐Y360D mutant inhibited the levels of *CLK4* mRNA (Figure S4A,B), suggesting that phosphorylated MITF has a different inhibitory role in regulating *CLK4*.

Together, MITF is a transcriptional suppressor of *CLK4* in ESCC.

### 3.11 Methionine oxidation at M307 impairs the activity of CLK4 and confers an overall proliferative and metabolic advantage to ESCC cells

**FIGURE 11:**
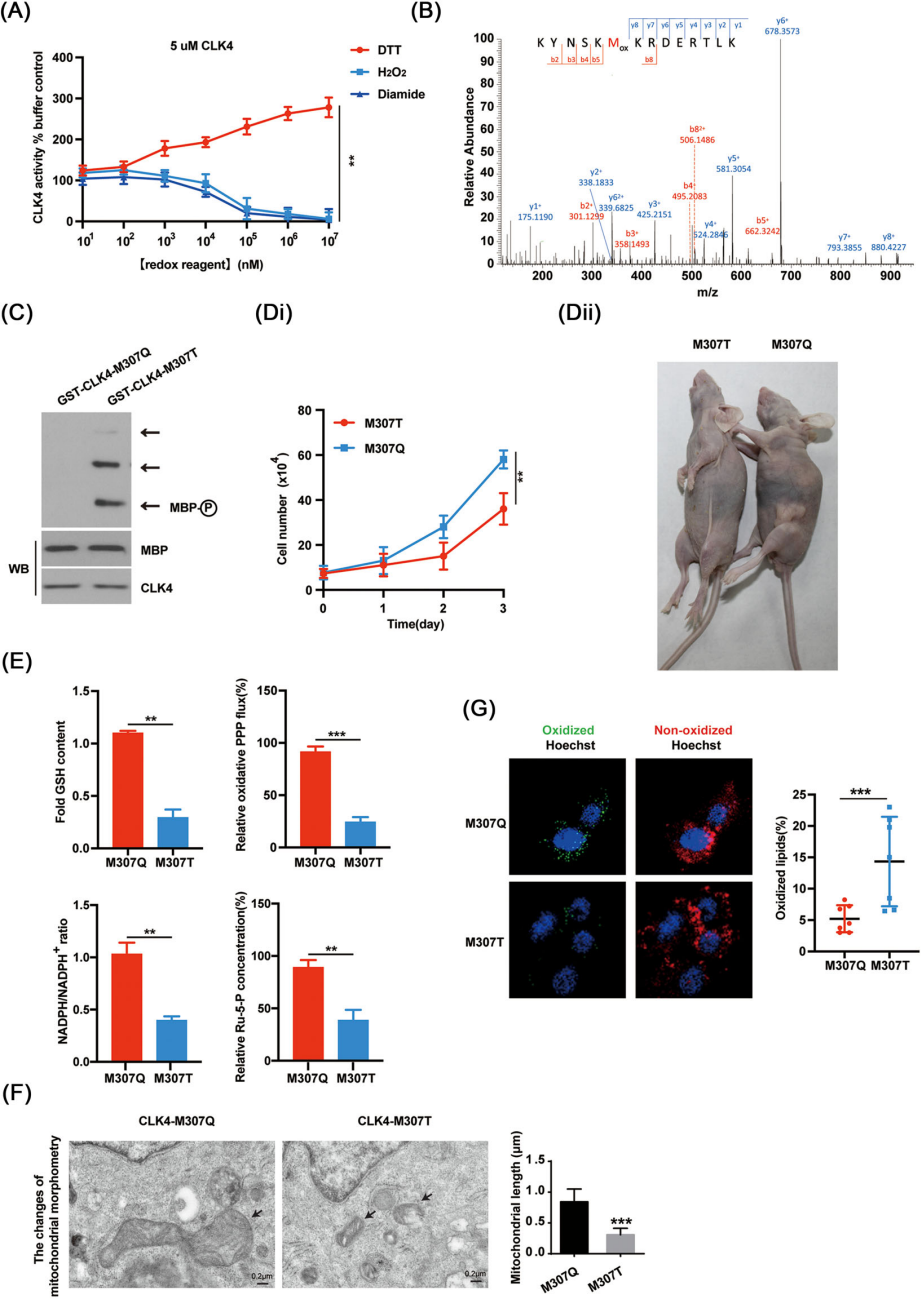
M307 oxidation impairs the activity of CDC‐like kinase 4 (CLK4) and confers an overall proliferative and metabolic advantage to esophageal squamous cell carcinoma (ESCC) cells. (A) The effects of H_2_O_2_, dithiothreitol (DTT) and diamide on the kinase activity of recombinant CLK4 protein by analysing the phosphorylation of the fluorescent peptide substrate. (B) Mass spectrometry (MS/MS) analysis was performed to identify the oxidized residues of CLK4. (C) The effects of *CLK4‐M307Q* and *CLK4‐M307T* on the phosphorylation of MBP. (Di) The effects of *CLK4‐M307Q* and *CLK4‐M307T* reintroduction on the proliferation of ESCC cells. (Dii) Xenograft mice were made using ESCC cells with *CLK4‐M307Q* and *CLK4‐M307T* reintroduction (*n* = 6/group). The representative images of the tumor were shown. (Ei‐v) The effects of *CLK4‐M307Q* and *CLK4‐M307T* reintroduction on the intermediates of PPP, GSH, NADPH/NADP^+^ and nucleotide metabolism pathways in ESCC cells. (F) The effects of *CLK4‐M307Q* and *CLK4‐M307T* reintroduction on the length of mitochondria in ESCC cells. (G) The effects of *CLK4‐M307Q* and *CLK4‐M307T* reintroduction on lipid peroxidation of ESCC cells. The above results were from three repeated experiments

Cellular redox status affects protein activity via oxidizing cysteine (C) or methionine (M) residues.^30^ Interestingly, H_2_O_2_ or Diamide (a thiol oxidizing agent) inhibited the activity of CLK4 in a dose‐dependent manner, while the reductant DTT had the opposite effect (Figure 11A), indicating that the catalytic activity of CLK4 is reversibly regulated by oxidation. To identify potential redox‐sensitive residues in CLK4, we performed MS/MS analysis, and the conserved M307 residue was identified as a potential target (Figure 11B). Compared with an oxidation‐resistant CLK4‐M307T mutant, mimicking oxidation of CLK4‐M307Q did not affect the phosphorylation of myelin basic protein (MBP), a physiological CLK4 target (Figure 11C), confirming that M307 oxidation inhibits CLK4 activity.

To clarify the clinical significance of M307 oxidation of CLK4 in ESCC cells, *CLK4‐M307T* and *CLK4‐M307Q* mutants were reintroduced into ESCC cells, respectively. It was observed that the reintroduction of *CLK4‐M307Q* in *CLK4* knockdown ESCC cells significantly enhanced cell proliferation and tumor growth in xenograft experiments as compared with the *CLK4‐M307T* group (Figure 11Di,ii). The metabolic profiling indicated that ESCC cells expressing *CLK4‐M307Q* exhibited higher GSH, oxidative PPP flux, NADPH/NADP^+^ ratio, ribulose‐5‐phosphate (Ru‐5‐P), the biosynthesis of RNA and DNA, and longer mitochondria, but less lipid peroxidation than the cells expressing *CLK4‐M307T* (Figure 11Ei‐v,F,G). Therefore, M307 oxidation impairs the activity of CLK4 and confers an overall proliferative and metabolic advantage to ESCC cells.

### 3.12 CLK4 is negatively associated with the expression of MITF in ESCC

To corroborate the observation in our mouse model, we examined CLK4 and MITF expression in human ESCC specimens. Analysis of human ESCC samples demonstrated that low CLK4 expression was related to high MITF and purine metabolic markers (Figure S5A,B). Furthermore, higher MITF and lower CLK4 in ESCC were significantly correlated with a poor OS (Figure S5C). These data reveal that CLK4‐MITF inverse regulation in ESCC was associated with patients’ survival.

## 4 DISCUSSION

In this study, we reveal a novel action mechanism of CLK4 in ESCC as a tumor inhibitor. We provide the following evidence: (1) CLK4 is markedly down‐regulated in ESCC cells and clinical samples, which is associated with the methylation of its promoter. (2) Enrichment of gene function from TCGA database and metabolic profiling of ESCC cells indicate that CLK4 in ESCC affects NADPH and purine synthesis pathway. (3) CLK4 inhibits ESCC development by blocking MITF‐enhanced nucleotide metabolism and redox homeostasis. (4) Mechanistically, WT‐CLK4, not kinase‐dead CLK4‐K189R mutant, directly phosphorylates MITF at Y360. This phosphorylation promotes its interaction with E3 ligase COP1 and its K63‐linked ubiquitination at K308/K372, which leads to SQSTM1 recognition and its selective autophagy degradation. (5) ZRANB1 rather than USP13 reverts the COP1‐mediated MITF ubiquitination and autophagy degradation. (6) MITF‐Y360F mutant has the opposite effects. (7) *MITF* binds to E‐ rather than M‐boxes in the promoter of *CLK4* and transcriptionally down‐regulates CLK4 expression in ESCC. (8) CLK4 itself is a redox‐sensitive kinase, and its methionine oxidation at M307 impairs kinase activity and contributes to ESCC. (9) Clinically, significant correlations are observed between low levels of CLK4 and increased MITF and purine metabolic markers in different pathological features, which predicts a poor clinical outcome of ESCC patients. Together, this study highlights the potential role of CLK4 oxidation at M307 residue in modulating the purine synthesis pathway and redox status in ESCCs, suggesting potential therapeutic targets in ESCC treatment.

One of the crucial discoveries in the current project is that we identify a previously unknown mechanism of CLK4 in the tumors, by which the CLK4‐MITF feedback axis sustains nucleic acid synthesis and NADPH level in ESCC. This new finding is consistent with recent reports that various kinases play important roles as the critical regulators of purine metabolism reprogramming in different tumors. For instance, 6‐phosphofructo‐2‐kinase/fructose‐2,6‐bisphosphatase 4, a glycolysis enzyme, increases pools of purines for breast cancer growth by activating steroid receptor coactivator‐3.^31^ Dual‐specificity tyrosine (Y) phosphorylation‐regulated kinase (DYRK) 3 reprograms purine synthesis by blocking NCOA3/ATF4 TF complex in hepatocellular carcinoma.^16^ U2AF homology motif kinase 1 enhances the de novo purine synthesis pathway, thereby promoting gastric cancer development.^19^ CLK3 activates the c‐Myc‐mediated transcription of purine metabolic genes by phosphorylating USP13 at Y708 in cholangiocarcinoma.^7^ ERK2 can phosphorylate T619 phosphorylation of phosphoribosylformylglycinamidine synthase in rat sarcoma viral oncogene (RAS)‐driven cancer cells to affect the activity of critical purine enzyme.^32^ We believe that future studies will identify more unknown kinases contributing to purine metabolic reprogramming and explore their potential therapeutic benefits in diverse tumor types. Notably, unlike CLK3 as an oncogene in CCA,^7^ CLK4 is demonstrated to act as a tumor inhibitor and disrupt redox homeostasis in ESCC. This difference is reminiscent of the DYRK kinase family, in which DYRK1A usually acts as an oncogene, while DYRK1B and DYRK3 inhibit tumor progression.^16^, ^33^ Given that DYRK3 loss contributes to purine synthesis,^16^ it may be valuable to explore other family members in reprogramming nucleotide metabolism and redox status in tumors.

Autophagy has been well‐known to be evolutionarily conserved across the eukaryotes and crucial for maintaining cellular homeostasis. Therefore, via autophagy, cells and organs can survive and protect against stress or adverse circumstances, including hypoxia, inflammation, starvation and radiotherapy.^34^ It has been well‐known that post‐translational modifications such as phosphorylation, acetylation and ubiquitination regulate autophagy induction.^12^ Although phosphorylation, acetylation and ubiquitination have been shown to finely tune the expression, stabilization and function of MITF,^35^ there is no report about MITF selective autophagy. Herein, we indicate that MITF phosphorylation at Y360 stimulates its selective autophagic degradation. It has been reported that a critical step in selective degradation is the binding of the autophagy receptors to ubiquitinated target proteins.^24^ This study shows that the phosphorylated MITF displays a stronger interaction with the COP1 E3 ligase, leading to its ubiquitination and recognition by SQSTM1. Notably, we do not explore the accurate mechanism of phosphorylation in enhancing the association of MITF with COP1 presently. However, we assume that phosphorylation of Y360 may result in the change in the conformation of MITF, which is akin to other proteins regulated via post‐translational modification shown in the literature.^7^ Thus, the phosphorylation at Y360 makes MITF accessible and subsequently recognized by COP1. Therefore, this finding proves that the crosstalk between phosphorylation and ubiquitination regulates MITF selective degradation in ESCC. In addition, although autophagy programs metabolic and functional fitness, this is the first report about its effects on purine synthesis.

The third important finding is that CLK4 itself is shown to be a redox‐sensitive kinase, and its methionine oxidation at M307 impairs kinase activity and contributes to ESCC.

These exciting data are consistent with the previous reports that oxidation usually impairs the activity of kinase.^36^ Furthermore, when we analysed the oxidation of CLK4, K308 ubiquitination of CLK4 was also identified. Therefore, it is tempting to reason that CLK4 oxidation at M307 may affect the ubiquitination of K308 site and protein stabilization. Of course, further study is needed to test this hypothesis. In addition, we also should clarify the enzyme that induces the oxidation of CLK4. The answers to these questions will let us know more about the effects of ROS on CLK4 functions in ESCC.

## 5 CONCLUSIONS

In summary, this study reveals that the crosstalk between CLK4 and MITF affects the malignant properties of ESCC cells (Figure S5D). This previously unrecognized finding will present new insight into the progression of ESCC, which will be helpful to the improvement of therapeutics.

## CONFLICT OF INTEREST

The authors declare that there is no conflict of interest that could be perceived as prejudicing the impartiality of the research reported.

## DATA ACCESSIBILITY STATEMENT

All data generated or analyzed during this study are included in this published article and its supplementary information files.

## Supporting information

Supporting Information

Supporting Information

Supporting Information

Supporting Information

Supporting Information

Supporting Information
