# Supplementary figures and images for: Methionine oxidation of CLK4 promotes the metabolic switch and redox homeostasis in esophageal carcinoma via inhibiting MITF selective autophagy
Source: Clin Transl Med. 2022 Jan 29;12(1):e719. doi: 10.1002/ctm2.719 (PMC8800482; doi:10.1002/ctm2.719)

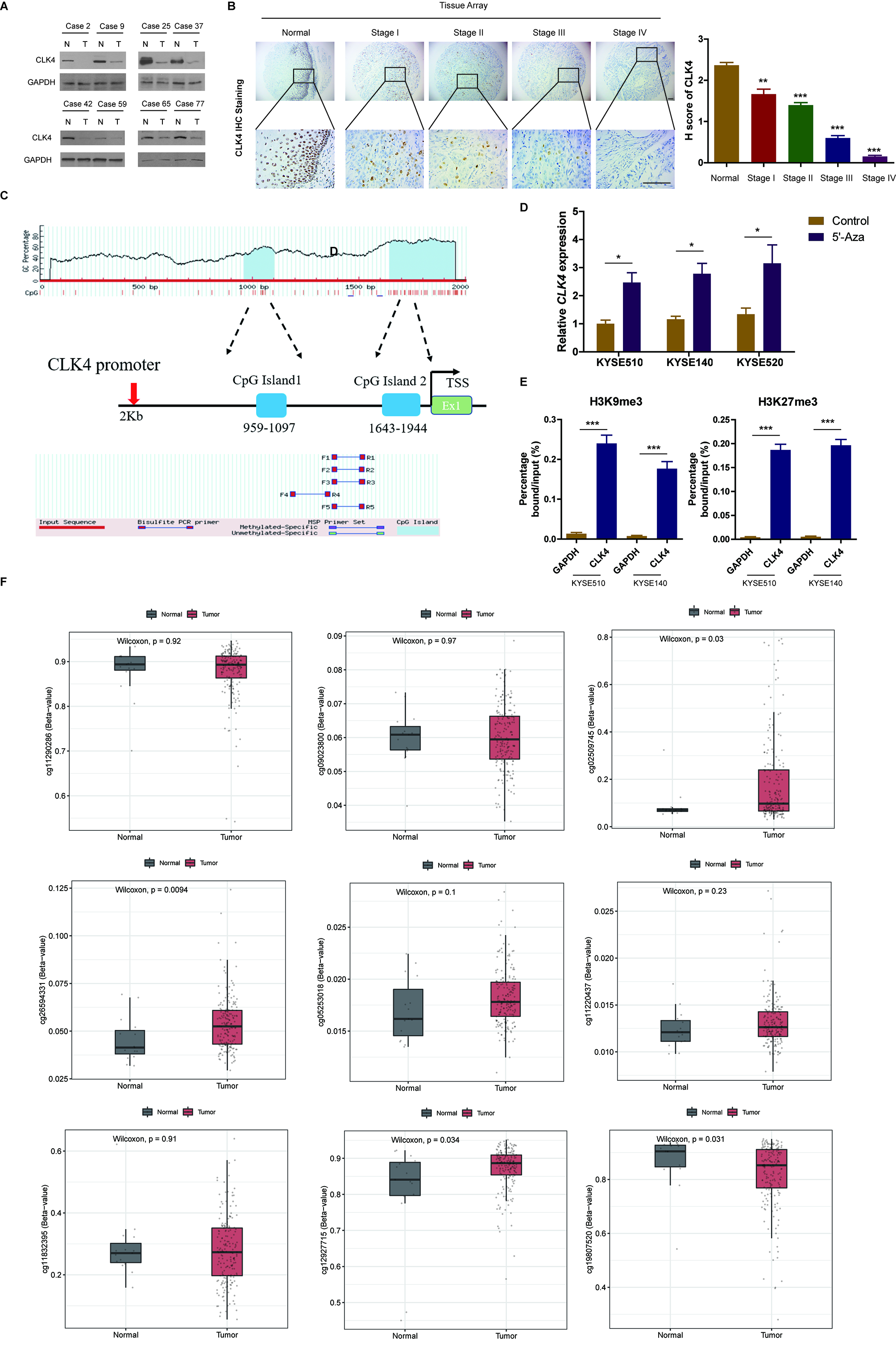

Supplement: Supplementary file 1 — Supporting Information [file CTM2-12-e719-s002.tif]

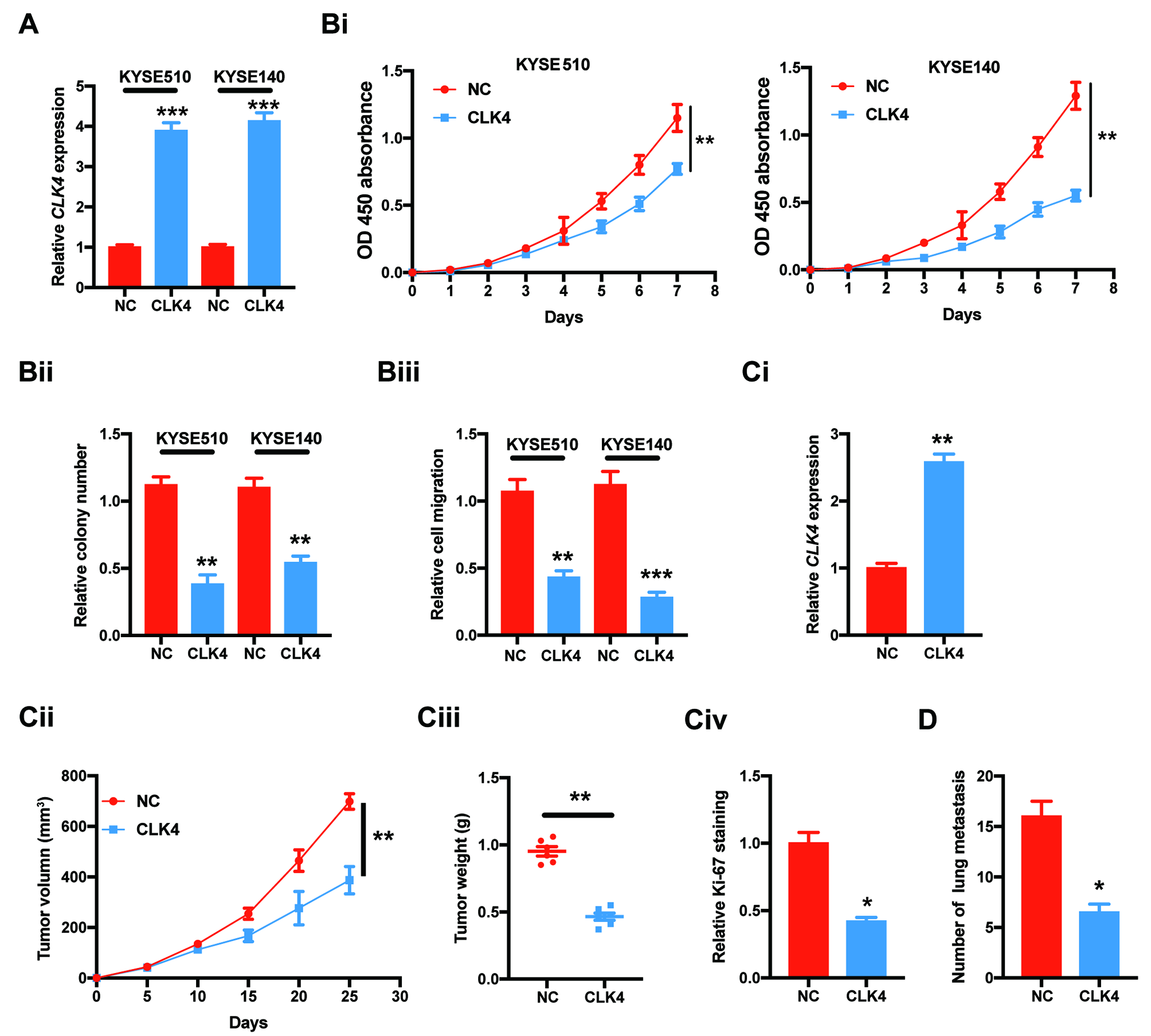

Supplement: Supplementary file 2 — Supporting Information [file CTM2-12-e719-s003.tif]

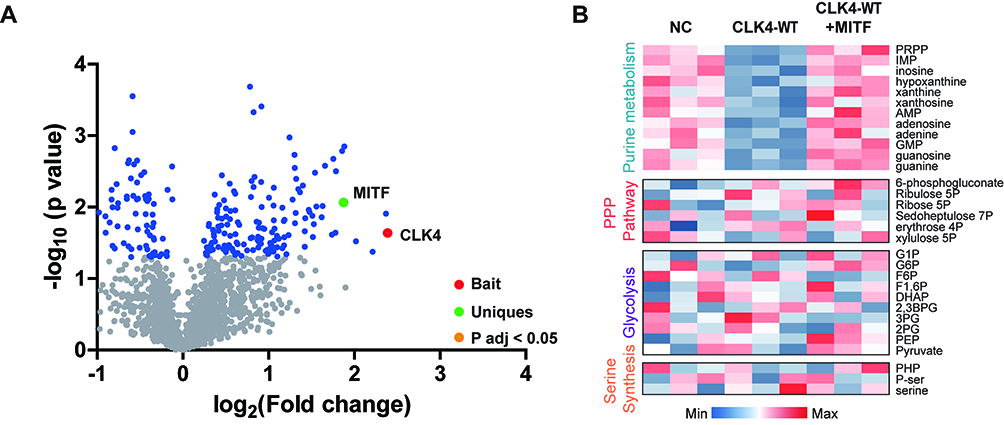

Supplement: Supplementary file 3 — Supporting Information [file CTM2-12-e719-s006.tif]

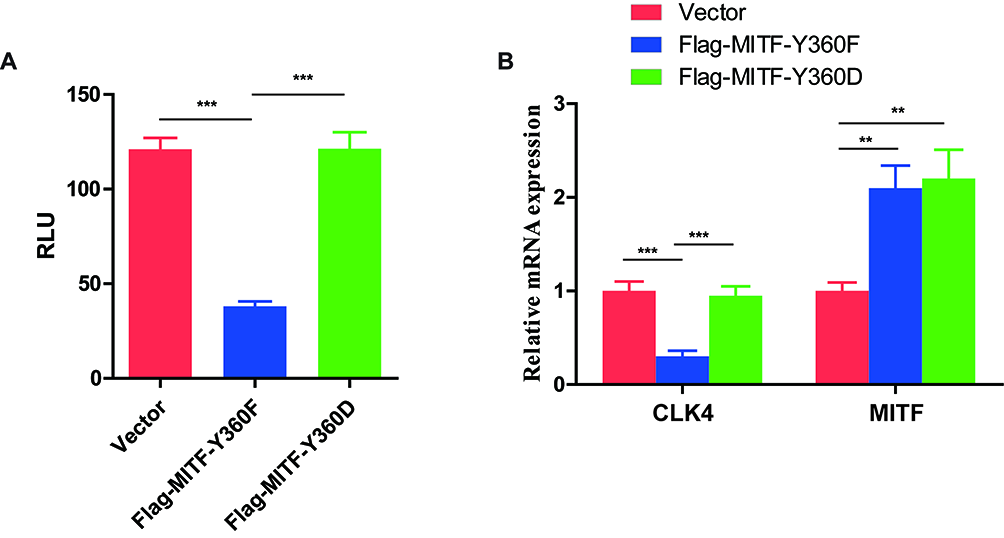

Supplement: Supplementary file 4 — Supporting Information [file CTM2-12-e719-s004.tif]

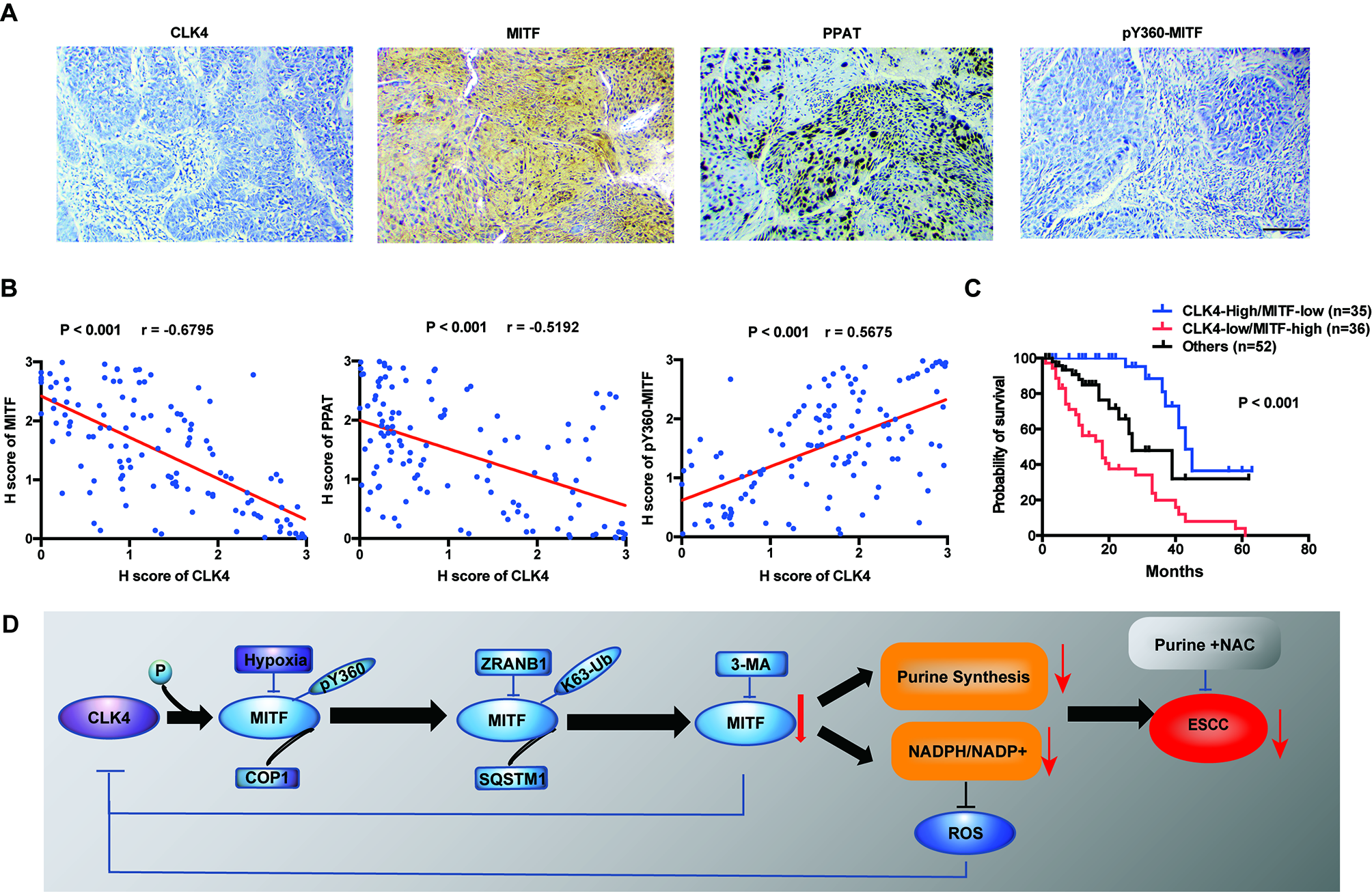

Supplement: Supplementary file 5 — Supporting Information [file CTM2-12-e719-s001.tif]
