# Supplementary material for: Methionine oxidation of CLK4 promotes the metabolic switch and redox homeostasis in esophageal carcinoma via inhibiting MITF selective autophagy
Source: Clin Transl Med. 2022 Jan 29;12(1):e719. doi: 10.1002/ctm2.719 (PMC8800482; doi:10.1002/ctm2.719)
Supplement: Supplementary file 6 — Supporting Information [file CTM2-12-e719-s005.docx]

**Supplementary figure legends**

**Figure S1. Downregulation** **of CLK4 expression explicitly predicts the poor outcomes of aggressive ESCC.** (**A**) Western blots were used to observe the levels of CLK4 in patients’ tissues with ESCC and control tissues. (**B**) CLK4 expression in the ESCC tissue arrays (n = 120) using IHC. Bar = 100μm. (**C**) The methylation profiling was examined in the CpG units of *CLK4* promoter region in TCGA normal (n = 16) and ESCA patients (n = 185). (**D**) ESCC cell lines were treated with 5 μM 5’-Aza for 96 hours. *CLK4* mRNA levels were examined by qRT-PCR. (**E**) ChIP studies in ESCC cells revealed that the *CLK4* promoter was enriched with H3K9me3 and H3K27me3. Immunoprecipitated DNA was quantified by qRT-PCR. GAPDH was used as a negative control. (**F**) The methylation status of other 9 CpG sites within *CLK4* promoter in TCGA datasets was respectively shown.

**Figure S2. Transfection of *CLK4* into KYSE510 and KYSE140 cells inhibits the malignant state of ESCC cells.** **(A)** The upregulation of *CLK4* was examined by qRT-PCR in KYSE510 and KYSE140 cell lines. (**Bi**) The effects of *CLK4* overexpression on KYSE510 and KYSE140 cell lines were examined using CCK-8 Kits. (**Bii**) The effects of *CLK4* overexpression on KYSE510 and KYSE140 cell lines on colony formation. (**Biii**) The effects of *CLK4* overexpression on the migration of KYSE510 and KYSE140 cells. (**Ci**)The levels of *CLK4* mRNA were measured in the tissues of dissected tumors using qRT-PCR. (**Cii**) The volumes of the tumor were shown in a time-course manner. And *CLK4* level in KYSE510 cells was stably upregulated. (**Ciii**) Tumor weight was shown as indicated. (**Civ**) The effects of CLK4 overexpression on tumor Ki67 staining. (**D**) The effects of CLK4 overexpression on lung metastasis of ESCC. The nodule number of lung metastasis was shown.

**Figure S3. MITF is a bona fide interactor of CLK4 and reverts the decrease in purine synthesis in CLK4‐upregulated ESCC cells. (A)**Volcano plot showing CLK4 interactors identified in KYSE140 cells with Empty-BioID2 or CLK4-BioID2. (**B**) Heatmap showing the effects of MITF on the metabolites of KYSE140 cells, which were examined by LC‐MS/MS.

**Figure S4. Phosphorylated MITF fails to binds and transcriptionally inhibits the promoter of *CLK4*. (A)** HEK293T cells were transfected with Flag-MITF-Y360F mutant or Flag-MITF-Y360D phosphorylation mimic mutant with a luciferase reporter of *CLK4*-luc, respectively. The relative luciferase activity (RLU) was examined. (**B**) ESCC cells were transfected with the indicated plasmids. The levels of *CLK4* mRNA were examined.

**Figure S5. CLK4 is negatively associated with the expression of MITF in ESCC.** (A) The expression of CLK4, MITF, pY360-MITF, and PPAT in human ESCC specimens. Bar = 100 μm. (B) The coefficient analysis of Pearson correlation about CLK4, pY360-MITF, MITF, and PPAT in human ESCC (n = 123). (C) Assays of Kaplan-Meier according to the expression of MITF and CLK4 in human ESCC. (D) Schematic model for the role of CLK4-MITF feedback axis in ESCC progression.

Table S1.

Top 550 up-regulated genes with low CLK4 expression

| Gene name | FDR |
| --- | --- |
| CLK4 | 3.8939E-42 |
| CLK1 | 2.6083E-23 |
| SEC31B | 1.1539E-18 |
| C5orf41 | 4.778E-17 |
| FAM13AOS | 2.0522E-16 |
| RPL21P44 | 3.476E-16 |
| SFRS18 | 4.3586E-16 |
| N4BP2L1 | 1.1566E-15 |
| CROCCL2 | 1.7938E-15 |
| CG030 | 2.0844E-15 |
| MALAT1 | 1.3349E-14 |
| ARGLU1 | 1.3699E-14 |
| TRIM78P | 4.2662E-14 |
| SFRS12 | 4.4178E-14 |
| UBQLNL | 5.0711E-14 |
| ABCA10 | 5.7455E-14 |
| KLHDC1 | 6.1174E-14 |
| N4BP2L2 | 6.7883E-14 |
| ZNF354B | 1.0471E-13 |
| NCRNA00201 | 1.5311E-13 |
| TRIM52 | 1.6211E-13 |
| ARGFXP2 | 2.2298E-13 |
| LRP2BP | 3.1359E-13 |
| SFRS11 | 3.4874E-13 |
| LENG8 | 4.0636E-13 |
| CNOT6L | 7.6678E-13 |
| KCNJ13 | 9.3745E-13 |
| LOC646471 | 1.0731E-12 |
| RNPC3 | 1.105E-12 |
| CCDC30 | 1.2721E-12 |
| ARID4A | 1.4456E-12 |
| ZNF75D | 1.519E-12 |
| NCRNA00182 | 1.9828E-12 |
| C6orf163 | 1.996E-12 |
| MFSD8 | 2.0288E-12 |
| RPS15AP10 | 3.0342E-12 |
| DKFZP586I1420 | 3.2534E-12 |
| SFRS5 | 3.437E-12 |
| IKZF5 | 3.5474E-12 |
| FAM193B | 4.0048E-12 |
| NKTR | 5.3658E-12 |
| PCMTD1 | 5.7221E-12 |
| C8orf79 | 5.9985E-12 |
| C6orf164 | 7.7634E-12 |
| ZNF441 | 7.8448E-12 |
| OGT | 8.1986E-12 |
| ASB14 | 9.0796E-12 |
| CTSO | 1.0561E-11 |
| TXNDC6 | 1.0693E-11 |
| MDM4 | 1.3278E-11 |
| C5orf56 | 1.358E-11 |
| SLC25A27 | 1.4975E-11 |
| RUFY3 | 1.852E-11 |
| CSAD | 2.1849E-11 |
| ALS2CR8 | 2.3996E-11 |
| RBM43 | 3.1943E-11 |
| RNF146 | 3.2502E-11 |
| C11orf61 | 3.4664E-11 |
| TTLL3 | 4.0334E-11 |
| INE1 | 4.1889E-11 |
| PPAT | 4.3941E-11 |
| ABCA6 | 4.4076E-11 |
| PWWP2A | 4.5399E-11 |
| LOC100272146 | 4.6579E-11 |
| GOLGA6L10 | 4.7487E-11 |
| ZFP2 | 5.2858E-11 |
| RBM39 | 6.0444E-11 |
| MGEA5 | 6.0476E-11 |
| AMY2B | 6.6389E-11 |
| ZC3H6 | 7.8667E-11 |
| ANKRD12 | 8.6208E-11 |
| AHSA2 | 9.7205E-11 |
| AGXT2L2 | 9.8989E-11 |
| C1orf152 | 1.1886E-10 |
| LUC7L3 | 1.5584E-10 |
| ZBTB1 | 1.7399E-10 |
| C5orf45 | 1.8279E-10 |
| ZNF211 | 1.8689E-10 |
| ZNF224 | 1.9713E-10 |
| PRPP | 2.0977E-10 |
| ABCA9 | 2.1161E-10 |
| ACVR2A | 2.3233E-10 |
| PAPD4 | 2.3778E-10 |
| NPHP3 | 2.4151E-10 |
| LOC100190986 | 2.4972E-10 |
| TRIM23 | 2.6535E-10 |
| LOC388692 | 2.792E-10 |
| PRPF39 | 2.8337E-10 |
| PPM1K | 2.848E-10 |
| OMG | 3.1938E-10 |
| ANKAR | 3.3591E-10 |
| PAN3 | 3.4952E-10 |
| ABCA5 | 3.5134E-10 |
| PRO0628 | 3.5214E-10 |
| LOC145474 | 4.1435E-10 |
| C1orf63 | 4.3376E-10 |
| ZNF546 | 4.395E-10 |
| MAPK8IP3 | 4.4083E-10 |
| FAM160B1 | 5.153E-10 |
| CD160 | 5.6133E-10 |
| MBD5 | 5.8764E-10 |
| USP53 | 5.888E-10 |
| VAMP2 | 6.1468E-10 |
| RCHY1 | 6.8069E-10 |
| ARID4B | 6.9125E-10 |
| DMXL1 | 7.5134E-10 |
| LUC7L | 7.8932E-10 |
| SYTL3 | 7.949E-10 |
| ZNF432 | 8.0098E-10 |
| C5orf53 | 8.0808E-10 |
| GPBP1 | 8.5813E-10 |
| ZNF154 | 9.4361E-10 |
| ZNF37B | 9.4915E-10 |
| ALPK1 | 9.5667E-10 |
| BTBD18 | 1.0033E-09 |
| C1orf175 | 1.1568E-09 |
| MLANA | 1.1786E-09 |
| ZMAT1 | 1.179E-09 |
| MRS2P2 | 1.2251E-09 |
| LOC91316 | 1.3065E-09 |
| LOC648740 | 1.3151E-09 |
| SPDYE1 | 1.3193E-09 |
| C6orf201 | 1.3478E-09 |
| C17orf57 | 1.3537E-09 |
| ELF2 | 1.371E-09 |
| BOD1L | 1.3917E-09 |
| MPHOSPH8 | 1.5099E-09 |
| SAP30L | 1.6848E-09 |
| C21orf62 | 1.6915E-09 |
| KLRA1 | 1.8329E-09 |
| PRRT2 | 1.8504E-09 |
| KIAA1109 | 1.8755E-09 |
| LOC100272228 | 1.8831E-09 |
| FLJ10213 | 1.9833E-09 |
| GNRH1 | 2.088E-09 |
| TAS2R4 | 2.1059E-09 |
| CALCOCO1 | 2.2057E-09 |
| YTHDC1 | 2.3455E-09 |
| RBM5 | 2.3849E-09 |
| ZNF182 | 2.3986E-09 |
| TAS2R10 | 2.4424E-09 |
| WDR52 | 2.569E-09 |
| CD69 | 2.6363E-09 |
| LRIT3 | 2.639E-09 |
| MIER1 | 2.6752E-09 |
| BCL-2 | 2.7217E-09 |
| FAM196A | 2.7304E-09 |
| NTRK3 | 2.8605E-09 |
| PCBD2 | 2.8951E-09 |
| C3orf48 | 3.0642E-09 |
| METTL14 | 3.1016E-09 |
| FBXW7 | 3.1046E-09 |
| IL11RA | 3.1495E-09 |
| ZNF814 | 3.1869E-09 |
| TIA1 | 3.402E-09 |
| C2orf60 | 3.5033E-09 |
| C2orf58 | 3.6439E-09 |
| TDRD6 | 3.9803E-09 |
| LOC728989 | 4.249E-09 |
| CDRT4 | 4.6952E-09 |
| REC8 | 4.8114E-09 |
| GART | 5.0712E-09 |
| LARP7 | 5.2704E-09 |
| MASP2 | 5.3309E-09 |
| AGAP5 | 5.3465E-09 |
| ANKDD1A | 5.4859E-09 |
| LOC90834 | 5.542E-09 |
| NPAS3 | 5.7872E-09 |
| TXK | 5.9027E-09 |
| SLC26A7 | 5.9549E-09 |
| ZNF767 | 6.0017E-09 |
| B3GALT2 | 6.2944E-09 |
| SFRS12IP1 | 6.6893E-09 |
| ZNF514 | 6.77E-09 |
| ZNF540 | 6.9555E-09 |
| ZNF226 | 7.1505E-09 |
| MAPKSP1 | 7.4764E-09 |
| FNBP4 | 7.8619E-09 |
| ZFP14 | 8.9345E-09 |
| CXorf23 | 9.0531E-09 |
| PTAR1 | 9.2488E-09 |
| BDH2 | 9.2872E-09 |
| RAPGEF2 | 9.8631E-09 |
| RAB33B | 9.9017E-09 |
| NFAT5 | 1.0204E-08 |
| ANKRD36 | 1.119E-08 |
| ZNF782 | 1.2028E-08 |
| TUBA3D | 1.2393E-08 |
| ADSL | 1.2591E-08 |
| DNAJB14 | 1.3028E-08 |
| ZRANB2 | 1.3081E-08 |
| LOC100128842 | 1.3123E-08 |
| GFRA1 | 1.3159E-08 |
| PTCH2 | 1.3188E-08 |
| ZNF449 | 1.3343E-08 |
| LOC100131434 | 1.3567E-08 |
| C14orf28 | 1.3699E-08 |
| KIAA0831 | 1.4315E-08 |
| LOC100132247 | 1.4546E-08 |
| CARD8 | 1.4714E-08 |
| ZNF776 | 1.55E-08 |
| NEAT1 | 1.5571E-08 |
| CCDC66 | 1.5775E-08 |
| OGN | 1.7161E-08 |
| IPCEF1 | 1.7364E-08 |
| APBB3 | 1.7829E-08 |
| ZNF836 | 1.7914E-08 |
| ATRX | 1.8002E-08 |
| POLI | 1.8179E-08 |
| SEPT7P2 | 1.8254E-08 |
| ZNF354A | 1.8707E-08 |
| POLK | 1.963E-08 |
| ABI3BP | 1.9862E-08 |
| PRO1768 | 1.9954E-08 |
| CCNL2 | 2.012E-08 |
| LOC148696 | 2.0508E-08 |
| TSSK4 | 2.0531E-08 |
| PRPF38B | 2.1006E-08 |
| LOC100128288 | 2.1533E-08 |
| BTAF1 | 2.2397E-08 |
| ZNF404 | 2.2776E-08 |
| GPRASP1 | 2.2909E-08 |
| C1QTNF7 | 2.4341E-08 |
| ZBTB16 | 2.4404E-08 |
| CDKL3 | 2.4486E-08 |
| C5orf44 | 2.4537E-08 |
| RGPD3 | 2.4641E-08 |
| KIAA1407 | 2.483E-08 |
| LOC100170939 | 2.5456E-08 |
| SPDYA | 2.6502E-08 |
| DNAJC27 | 2.6681E-08 |
| WSB1 | 2.6877E-08 |
| CENPC1 | 2.8595E-08 |
| PTGIS | 2.9711E-08 |
| CEP120 | 3.0051E-08 |
| C5orf4 | 3.0426E-08 |
| RUNDC2C | 3.0432E-08 |
| ZNF708 | 3.0451E-08 |
| SPDYE8P | 3.0492E-08 |
| SNORD116-28 | 3.1117E-08 |
| RASA4P | 3.144E-08 |
| ZNF204P | 3.2108E-08 |
| RSBN1 | 3.2262E-08 |
| COLQ | 3.307E-08 |
| SDHAP3 | 3.3552E-08 |
| ZDHHC17 | 3.3688E-08 |
| DDX17 | 3.4352E-08 |
| CLNK | 3.4728E-08 |
| ZNF491 | 3.4852E-08 |
| ZNF862 | 3.4994E-08 |
| ZMYM6 | 3.575E-08 |
| MCART6 | 3.5763E-08 |
| GUSBP3 | 3.5868E-08 |
| GVIN1 | 3.6392E-08 |
| MYSM1 | 3.6548E-08 |
| LOC285593 | 3.7371E-08 |
| ZNF548 | 3.7704E-08 |
| C11orf34 | 3.7898E-08 |
| NRIP2 | 3.821E-08 |
| CUX2 | 3.8712E-08 |
| ZNF700 | 3.8746E-08 |
| MAPLC3 | 3.8903E-08 |
| USPL1 | 3.9308E-08 |
| MSTN | 3.9952E-08 |
| AKD1 | 4.022E-08 |
| SLC18A2 | 4.036E-08 |
| C4orf29 | 4.0685E-08 |
| BTNL9 | 4.138E-08 |
| CHAD | 4.1391E-08 |
| FKBP1AP1 | 4.1645E-08 |
| ALS2CR12 | 4.1784E-08 |
| MIER3 | 4.4074E-08 |
| NTN5 | 4.6684E-08 |
| ATP6AP1L | 4.7425E-08 |
| LOC729603 | 4.7494E-08 |
| GPR64 | 4.7595E-08 |
| ZNF841 | 4.7718E-08 |
| ECHDC2 | 4.7836E-08 |
| MTMR7 | 4.8271E-08 |
| OCLM | 5.0692E-08 |
| ITGA10 | 5.2518E-08 |
| ZFC3H1 | 5.378E-08 |
| APOD | 5.4108E-08 |
| ZNF586 | 5.4665E-08 |
| LOC200030 | 5.7384E-08 |
| ZBED5 | 5.9466E-08 |
| VPS15 | 5.9594E-08 |
| SPATA1 | 5.964E-08 |
| SH2B1 | 6.038E-08 |
| FAM18A | 6.0704E-08 |
| PPWD1 | 6.0845E-08 |
| LOC100188949 | 6.2937E-08 |
| RUNDC3B | 6.3112E-08 |
| GOLGA8B | 6.3152E-08 |
| ABCB1 | 6.4012E-08 |
| ZNF493 | 6.5331E-08 |
| FLJ13197 | 6.6967E-08 |
| SPCS3 | 6.6981E-08 |
| GPR18 | 6.778E-08 |
| THAP6 | 6.9327E-08 |
| RWDD3 | 7.1705E-08 |
| ZNF638 | 7.1847E-08 |
| BDP1 | 7.2878E-08 |
| CYLD | 7.3114E-08 |
| ZSWIM6 | 7.3248E-08 |
| INO80D | 7.3265E-08 |
| CYFIP2 | 7.8398E-08 |
| SCN11A | 7.953E-08 |
| HPX | 7.9537E-08 |
| FLJ45340 | 8.0277E-08 |
| DDX26B | 8.1045E-08 |
| C14orf148 | 8.1148E-08 |
| CIR1 | 8.1565E-08 |
| B3GAT1 | 8.1829E-08 |
| ?\|728788 | 8.2777E-08 |
| ZNF671 | 8.5032E-08 |
| ZNF280D | 8.6863E-08 |
| TAS2R5 | 8.7251E-08 |
| PHF10 | 8.737E-08 |
| ZNF248 | 8.7955E-08 |
| ZNF292 | 8.9049E-08 |
| NAIP | 8.9333E-08 |
| AFF3 | 8.9727E-08 |
| ZNF264 | 8.989E-08 |
| PLCXD3 | 9.0501E-08 |
| PPIG | 9.0548E-08 |
| MBNL2 | 9.0571E-08 |
| RS1 | 9.4195E-08 |
| DLG2 | 9.4731E-08 |
| BRD7P3 | 9.5304E-08 |
| C2orf63 | 9.7436E-08 |
| GCFC1 | 9.7513E-08 |
| SLFN14 | 9.8485E-08 |
| DOPEY1 | 9.9948E-08 |
| ANKRD20A3 | 1.0249E-07 |
| FLT3 | 1.0597E-07 |
| FLJ36777 | 1.079E-07 |
| CACNB2 | 1.1018E-07 |
| TTC18 | 1.1135E-07 |
| RNF19A | 1.1362E-07 |
| C15orf28 | 1.1365E-07 |
| ARRDC5 | 1.1384E-07 |
| BMS1P5 | 1.1804E-07 |
| BBS12 | 1.1862E-07 |
| IL18R1 | 1.2037E-07 |
| ZNF346 | 1.2038E-07 |
| TP53INP1 | 1.2181E-07 |
| DLEU2L | 1.2388E-07 |
| PIKFYVE | 1.2473E-07 |
| CREBL2 | 1.2849E-07 |
| KIF3A | 1.2893E-07 |
| B3GAT2 | 1.2901E-07 |
| ATXN7L1 | 1.2927E-07 |
| BAZ2B | 1.3419E-07 |
| ABCA8 | 1.3587E-07 |
| LOC100132724 | 1.4092E-07 |
| H2BFXP | 1.4759E-07 |
| ANKRD20B | 1.485E-07 |
| RNF180 | 1.4877E-07 |
| CASD1 | 1.4879E-07 |
| C10orf118 | 1.4936E-07 |
| C15orf27 | 1.4977E-07 |
| ZNF721 | 1.5318E-07 |
| LOC202181 | 1.557E-07 |
| C2orf67 | 1.5701E-07 |
| FAM95B1 | 1.5813E-07 |
| CA5B | 1.6019E-07 |
| KLRC4 | 1.6062E-07 |
| ZNF44 | 1.6228E-07 |
| SYNPO2 | 1.6321E-07 |
| ARRDC3 | 1.6459E-07 |
| ?\|155060 | 1.6699E-07 |
| TXNIP | 1.7087E-07 |
| RCOR3 | 1.7553E-07 |
| PCDHB19P | 1.7615E-07 |
| ZNF225 | 1.7861E-07 |
| BRWD1 | 1.7919E-07 |
| ZNF674 | 1.8301E-07 |
| SEC1 | 1.8434E-07 |
| ATG16L1 | 1.851E-07 |
| TBX19 | 1.8646E-07 |
| PLCL1 | 1.8824E-07 |
| NCRNA00105 | 1.8848E-07 |
| CCDC144A | 1.9384E-07 |
| LYSMD3 | 1.9423E-07 |
| HSFX2 | 1.9549E-07 |
| RGS13 | 1.965E-07 |
| LOC441204 | 1.9716E-07 |
| LOC286367 | 1.9972E-07 |
| MOBKL1A | 2.0086E-07 |
| C10orf131 | 2.0351E-07 |
| C10orf32 | 2.0628E-07 |
| GPR171 | 2.0931E-07 |
| GPCPD1 | 2.0996E-07 |
| MGC16384 | 2.2535E-07 |
| LRRC70 | 2.2644E-07 |
| ZNF610 | 2.2784E-07 |
| RBM26 | 2.2992E-07 |
| C17orf69 | 2.3105E-07 |
| WDR60 | 2.3299E-07 |
| ERO1LB | 2.3339E-07 |
| SNED1 | 2.3923E-07 |
| SENP7 | 2.4085E-07 |
| KIAA0907 | 2.4248E-07 |
| ZFP62 | 2.4631E-07 |
| ZC3H13 | 2.4722E-07 |
| MAB21L2 | 2.5231E-07 |
| MPPE1 | 2.5836E-07 |
| PLA2G12A | 2.5927E-07 |
| TMEM161B | 2.6605E-07 |
| C10orf4 | 2.6808E-07 |
| LRRC39 | 2.703E-07 |
| EIF4E3 | 2.7298E-07 |
| RBM33 | 2.7357E-07 |
| RRN3P3 | 2.7464E-07 |
| CCDC39 | 2.7487E-07 |
| GDAP1L1 | 2.8064E-07 |
| PNPLA7 | 2.8834E-07 |
| ZNF250 | 2.8915E-07 |
| ZNF233 | 2.8952E-07 |
| NLGN3 | 2.9239E-07 |
| ZNF234 | 2.9394E-07 |
| RYBP | 2.955E-07 |
| SCN7A | 3.0181E-07 |
| ZNF791 | 3.0592E-07 |
| FAM13B | 3.0754E-07 |
| SQSTM1 | 3.1083E-07 |
| FLJ39653 | 3.1195E-07 |
| GABBR1 | 3.1477E-07 |
| ZNF337 | 3.1948E-07 |
| ZNF660 | 3.1949E-07 |
| ZNF808 | 3.2106E-07 |
| PCDHGA6 | 3.2181E-07 |
| CA3 | 3.2292E-07 |
| PKHD1L1 | 3.2562E-07 |
| ZNF230 | 3.275E-07 |
| TANK | 3.3057E-07 |
| TMEM100 | 3.4072E-07 |
| RGPD4 | 3.4403E-07 |
| FAM122C | 3.4444E-07 |
| RUBICON | 3.5715E-07 |
| CC2D2B | 3.5968E-07 |
| RRN3P1 | 3.6005E-07 |
| C7 | 3.635E-07 |
| RC3H1 | 3.6469E-07 |
| NANOG | 3.6656E-07 |
| ZNF506 | 3.6834E-07 |
| GIMAP7 | 3.6866E-07 |
| C17orf108 | 3.7935E-07 |
| PTPRM | 3.8363E-07 |
| CNOT8 | 3.8597E-07 |
| CCDC141 | 3.8639E-07 |
| CLCN6 | 3.8769E-07 |
| RNF138P1 | 3.9207E-07 |
| SYNE1 | 3.9445E-07 |
| ZNF615 | 3.9849E-07 |
| TSGA10 | 3.9909E-07 |
| DMTF1 | 3.997E-07 |
| PCGF3 | 4.086E-07 |
| ADC | 4.1449E-07 |
| FAM107A | 4.2276E-07 |
| PVRIG | 4.2281E-07 |
| ZNF805 | 4.3009E-07 |
| GCET2 | 4.3048E-07 |
| IL15 | 4.3999E-07 |
| VWA3A | 4.4026E-07 |
| CD302 | 4.4453E-07 |
| GRIA2 | 4.4912E-07 |
| GAPT | 4.5538E-07 |
| FAM22F | 4.6332E-07 |
| YPEL1 | 4.6445E-07 |
| PTBP2 | 4.687E-07 |
| FLJ42393 | 4.7393E-07 |
| DNAJB9 | 4.7922E-07 |
| C1orf168 | 4.9291E-07 |
| RGPD6 | 4.9732E-07 |
| CLEC4F | 5.007E-07 |
| P2RY12 | 5.1856E-07 |
| KCNQ1OT1 | 5.207E-07 |
| ZNF780B | 5.2279E-07 |
| ZNF23 | 5.257E-07 |
| ING3 | 5.2582E-07 |
| PPAP2A | 5.2921E-07 |
| FAM151A | 5.3092E-07 |
| C8orf34 | 5.4303E-07 |
| PUS10 | 5.4519E-07 |
| ATIC | 5.6346E-07 |
| NAPB | 5.6382E-07 |
| GRIA1 | 5.8233E-07 |
| ZCWPW2 | 6.371E-07 |
| MUSK | 6.379E-07 |
| ZNF397OS | 6.5532E-07 |
| PDXDC2 | 6.7227E-07 |
| RBM25 | 6.7267E-07 |
| SIGLEC6 | 6.7419E-07 |
| TCEANC | 6.8296E-07 |
| FRYL | 6.8988E-07 |
| ULK1 | 6.9912E-07 |
| PLGLB2 | 7.0271E-07 |
| RAB11FIP2 | 7.0429E-07 |
| RIC3 | 7.0799E-07 |
| LOC285830 | 7.1172E-07 |
| WDR27 | 7.1187E-07 |
| LOC100270804 | 7.1266E-07 |
| UTRN | 7.2159E-07 |
| ITSN2 | 7.2897E-07 |
| LRRC37B | 7.3689E-07 |
| C16orf52 | 7.5896E-07 |
| ZNF844 | 7.6929E-07 |
| ZNF235 | 7.8154E-07 |
| GPR133 | 7.8967E-07 |
| SAMD3 | 8.0295E-07 |
| HDC | 8.1105E-07 |
| IMPG1 | 8.1177E-07 |
| NALCN | 8.148E-07 |
| SOS1 | 8.1504E-07 |
| ZNF321 | 8.307E-07 |
| CCDC146 | 8.3384E-07 |
| PRO0611 | 8.4721E-07 |
| IKZF1 | 8.9868E-07 |
| KIAA0141 | 9.0026E-07 |
| KIAA0748 | 9.0097E-07 |
| BTLA | 9.1879E-07 |
| TSSK3 | 9.2316E-07 |
| KCNT2 | 9.2713E-07 |
| AFF1 | 9.2758E-07 |
| GHRLOS | 9.3205E-07 |
| ZNF564 | 9.3488E-07 |
| KCNIP1 | 9.3849E-07 |
| TAS2R20 | 9.4518E-07 |
| LOC162632 | 9.4992E-07 |
| LOC284440 | 9.5044E-07 |
| CMYA5 | 9.5179E-07 |
| P2RY14 | 9.585E-07 |
| CELF6 | 9.6045E-07 |
| ZNF429 | 9.6572E-07 |
| RHD | 9.7513E-07 |
| ATG16L2 | 9.8366E-07 |
| OFD1 | 9.8944E-07 |
| CCNT2 | 9.99E-07 |
| ZMYM5 | 1.0021E-06 |
| KCNIP2 | 1.0214E-06 |
| ZRSR2 | 1.029E-06 |
| ZC3H7A | 1.03E-06 |
| TAS2R14 | 1.0322E-06 |
| ZNF846 | 1.0327E-06 |
| NAALAD2 | 1.0348E-06 |
| LOC390595 | 1.0363E-06 |
| CPAMD8 | 1.0393E-06 |
| ZNF439 | 1.0408E-06 |

Table S2.

Mass spectrometry-based metabolic profiling of KYSE140 cells expressing NC, CLK4-WT with or without MITF.

|  | NC | NC | NC | CLK4-WT | CLK4-WT | CLK4-WT | CLK4-WT +MITF | CLK4-WT +MITF | CLK4-WT +MITF |
| --- | --- | --- | --- | --- | --- | --- | --- | --- | --- |
| PRPP | 1365.41 | 1289.6 | 1189.54 | 701.23 | 659.48 | 775.6 | 1469.5 | 1268.5 | 1689.8 |
| IMP | 4012.5 | 3689.7 | 4126.8 | 2103.6 | 1895.6 | 2563.3 | 3699.5 | 4001.25 | 3845.6 |
| inosine | 1895.66 | 1955.88 | 2196.5 | 1369.8 | 1456.6 | 1063.95 | 2016.56 | 2144.95 | 1822.64 |
| hypoxanthine | 7145.99 | 6598.25 | 6345.69 | 5126.87 | 4603.98 | 5545.6 | 5916.33 | 6598.47 | 6749.8 |
| xanthine | 5698.56 | 5126.58 | 5418.64 | 4562.85 | 4952.23 | 4215.55 | 5562.33 | 6048.9 | 5748.45 |
| xanthosine | 4596.85 | 4156.54 | 4322.84 | 3649.14 | 3458.45 | 3046.85 | 4485.75 | 3945.98 | 4231.52 |
| AMP | 11263.12 | 9856.45 | 9568.45 | 7986.98 | 8012.63 | 6512.35 | 9563.48 | 12456.25 | 10583.21 |
| adenine | 3269.52 | 3625.45 | 3452.12 | 1698.85 | 2015.56 | 2356.11 | 3954.56 | 3784.11 | 3563.96 |
| adenosine | 1965.56 | 2157.85 | 1895.65 | 1562.32 | 1695.89 | 1452.65 | 2012.56 | 2256.39 | 1848.96 |
| GMP | 1256.85 | 1658.44 | 1369.52 | 701.2 | 500.62 | 498.85 | 1455.85 | 1398.85 | 1741.21 |
| guanine | 4001.22 | 3569.42 | 3784.52 | 3006.95 | 3412.55 | 2985.65 | 4125.63 | 3985.56 | 4065.23 |
| guanosine | 2598.68 | 2156.39 | 2000.74 | 600.85 | 958.62 | 714.56 | 2341.56 | 2765.9 | 2558.63 |
|  |  |  |  |  |  |  |  |  |  |
| PHP | 4563.21 | 3956.25 | 4123.6 | 4326.5 | 3854.12 | 4021.36 | 3754.69 | 4321.56 | 4621.2 |
| P-ser | 2163.2 | 2845.62 | 3001.25 | 2784.65 | 1998.56 | 2985.65 | 3045.58 | 2365.9 | 2485.62 |
| serine | 8956.85 | 9562.3 | 7958.6 | 9412.6 | 8854.6 | 10658.4 | 9632.1 | 8521.6 | 9512.6 |
|  |  |  |  |  |  |  |  |  |  |
| G1P | 3362.5 | 3025.6 | 3562.1 | 3451.2 | 3748.2 | 3152.5 | 3789.3 | 3321.6 | 3654.8 |
| G6P | 5213.6 | 5869.4 | 5001.2 | 4569.8 | 4852.3 | 5128.6 | 5469.2 | 5785.2 | 5556.1 |
| F6P | 4896.5 | 4458.6 | 4589.1 | 4158.9 | 4785.9 | 4532.85 | 3996.8 | 4051.85 | 4258.6 |
| F1,6P | 2259.6 | 2541.2 | 2865.3 | 2741.56 | 2654.32 | 2584.25 | 3021.5 | 2596.3 | 2741.6 |
| DHAP | 1159.6 | 1542.3 | 1985.2 | 1745.6 | 1625.2 | 1485.6 | 1852.6 | 1258.6 | 1745.6 |
| 2,3BPG | 2589.6 | 2245.6 | 2016.3 | 2148.9 | 2365.8 | 2456.2 | 2256.8 | 2389.5 | 2014.56 |
| 3PG | 7489.56 | 7021.56 | 7269.6 | 7745.9 | 7532.1 | 7156.9 | 6986.5 | 7005.9 | 7123.6 |
| 2PG | 1584.5 | 1456.9 | 1320.5 | 1596.3 | 1425.6 | 1120.3 | 1056.3 | 1586.2 | 1402.3 |
| PEP | 3021.5 | 3369.2 | 3658.1 | 3541.2 | 3498.6 | 3367.8 | 4005.1 | 3785.2 | 3614.5 |
| Pyruvate | 18596.2 | 15893.6 | 20015.6 | 19856.2 | 17423.1 | 15556.3 | 20048.6 | 19365.1 | 18552.3 |
|  |  |  |  |  |  |  |  |  |  |
| 6-phosphogluconate | 812.3 | 774.8 | 800.2 | 811.2 | 833.6 | 812.6 | 824.56 | 853.2 | 834.6 |
| Ribulose 5P | 2745.29 | 3259.85 | 2861.22 | 700.21 | 658.15 | 674.36 | 2623.69 | 2695.23 | 3645.25 |
| Ribose 5P | 4002.3 | 3452.3 | 3741.25 | 968.56 | 973.21 | 985.62 | 3890.15 | 3885.62 | 3958.6 |
| Sedoheptulose 7P | 1653.63 | 1895.63 | 1742.56 | 1963.52 | 1685.36 | 1736.95 | 2105.69 | 1826.31 | 1756.98 |
| erythrose 4P | 735.6 | 652.36 | 702.56 | 725.85 | 733.69 | 741.23 | 698.58 | 730.25 | 717.56 |
| xylulose 5P | 3526.96 | 3369.51 | 3258.12 | 3005.69 | 3108.56 | 3405.86 | 2968.65 | 3207.8 | 3480.5 |
